# Whole Genome Deep Sequencing of HIV-1 Reveals the Impact of Early Minor Variants Upon Immune Recognition During Acute Infection

**DOI:** 10.1371/journal.ppat.1002529

**Published:** 2012-03-08

**Authors:** Matthew R. Henn, Christian L. Boutwell, Patrick Charlebois, Niall J. Lennon, Karen A. Power, Alexander R. Macalalad, Aaron M. Berlin, Christine M. Malboeuf, Elizabeth M. Ryan, Sante Gnerre, Michael C. Zody, Rachel L. Erlich, Lisa M. Green, Andrew Berical, Yaoyu Wang, Monica Casali, Hendrik Streeck, Allyson K. Bloom, Tim Dudek, Damien Tully, Ruchi Newman, Karen L. Axten, Adrianne D. Gladden, Laura Battis, Michael Kemper, Qiandong Zeng, Terrance P. Shea, Sharvari Gujja, Carmen Zedlack, Olivier Gasser, Christian Brander, Christoph Hess, Huldrych F. Günthard, Zabrina L. Brumme, Chanson J. Brumme, Suzane Bazner, Jenna Rychert, Jake P. Tinsley, Ken H. Mayer, Eric Rosenberg, Florencia Pereyra, Joshua Z. Levin, Sarah K. Young, Heiko Jessen, Marcus Altfeld, Bruce W. Birren, Bruce D. Walker, Todd M. Allen

**Affiliations:** 1 Broad Institute of MIT and Harvard, Cambridge, Massachusetts, United States of America; 2 Ragon Institute of MGH, MIT and Harvard, Boston, Massachusetts, United States of America; 3 HIV Clinic Praxis. Jessen, Berlin, Germany; 4 Immunobiology Lab, Department of Biomedicine, University Hospital Basel, Basel, Switzerland; 5 Institució Catalana de Recerca i Estudis Avançats (ICREA), Barcelona, Spain; 6 Irsicaixa AIDS Research Institute-HIVACAT, Hospital University Germans Trias I Pujol, Badalona, Spain; 7 Division of Infectious Diseases and Hospital Epidemiology, University Hospital Zurich, University of Zurich, Switzerland; 8 Massachusetts General Hospital and Harvard Medical School, Boston, Massachusetts, United States of America; 9 The Fenway Institute, Fenway Health, Boston, Massachusetts, United States of America; 10 Howard Hughes Medical Institute, Chevy Chase, Maryland, United States of America; Nationwide Children's Hospital, United States of America

## Abstract

Deep sequencing technologies have the potential to transform the study of highly variable viral pathogens by providing a rapid and cost-effective approach to sensitively characterize rapidly evolving viral quasispecies. Here, we report on a high-throughput whole HIV-1 genome deep sequencing platform that combines 454 pyrosequencing with novel assembly and variant detection algorithms. In one subject we combined these genetic data with detailed immunological analyses to comprehensively evaluate viral evolution and immune escape during the acute phase of HIV-1 infection. The majority of early, low frequency mutations represented viral adaptation to host CD8+ T cell responses, evidence of strong immune selection pressure occurring during the early decline from peak viremia. CD8+ T cell responses capable of recognizing these low frequency escape variants coincided with the selection and evolution of more effective secondary HLA-anchor escape mutations. Frequent, and in some cases rapid, reversion of transmitted mutations was also observed across the viral genome. When located within restricted CD8 epitopes these low frequency reverting mutations were sufficient to prime *de novo* responses to these epitopes, again illustrating the capacity of the immune response to recognize and respond to low frequency variants. More importantly, rapid viral escape from the most immunodominant CD8+ T cell responses coincided with plateauing of the initial viral load decline in this subject, suggestive of a potential link between maintenance of effective, dominant CD8 responses and the degree of early viremia reduction. We conclude that the early control of HIV-1 replication by immunodominant CD8+ T cell responses may be substantially influenced by rapid, low frequency viral adaptations not detected by conventional sequencing approaches, which warrants further investigation. These data support the critical need for vaccine-induced CD8+ T cell responses to target more highly constrained regions of the virus in order to ensure the maintenance of immunodominant CD8 responses and the sustained decline of early viremia.

## Author Summary

The ability of HIV-1 and other highly variable pathogens to rapidly mutate to escape vaccine-induced immune responses represents a major hurdle to the development of effective vaccines to these highly persistent pathogens. Application of next-generation or deep sequencing technologies to the study of host pathogens could significantly improve our understanding of the mechanisms by which these pathogens subvert host immunity, and aid in the development of novel vaccines and therapeutics. Here, we developed a 454 deep sequencing approach to enable the sensitive detection of low-frequency viral variants across the entire HIV-1 genome. When applied to the acute phase of HIV-1 infection we observed that the majority of early, low frequency mutations represented viral adaptations to host cellular immune responses, evidence of strong host immunity developing during the early decline of peak viral load. Rapid viral escape from the most dominant immune responses however correlated with loss of this initial viral control, suggestive of the importance of mounting immune responses against more conserved regions of the virus. These data provide a greater understanding of the early evolutionary events subverting the ability of host immune responses to control early HIV-1 replication, yielding important insight into the design of more effective vaccine strategies.

## Introduction

A major challenge to the development of effective vaccines against highly variable viruses is their ability to adapt to evade host immune responses [1]–[4]. During HIV-1 infection, for example, immune escape mutations develop which impair the ability of both CD8+ T cell responses and neutralizing antibodies to maintain immune control [5]–[9]. However, some CD8+ T cell escape mutations have been shown to dramatically impair viral replication capacity, which may slow viral escape and contribute significantly to the ability of some responses to effectively control HIV-1 [10]–[13]. The outcome of this dynamic interplay between immune responses functioning to eliminate infected cells, emerging escape variants that evade these responses, and the impact of these variants on viral replication, critically influences early immune control of HIV-1.

The majority of studies on HIV-1 evolution have relied on bulk Sanger sequencing to define the major genetic variants that arise during infection. These studies have demonstrated that upwards of 50% of mutations observed over the course of infection may be associated with viral adaptations to CD8+ T cell responses [5], [14]. Unfortunately, bulk Sanger sequencing is insufficient to detect low frequency variants that are particularly important during the acute phase of infection when viral escape occurs rapidly. The application of single genome amplification and sequencing (SGA or SGS) has increased the sensitivity for detecting and quantifying low frequency viral variants [7], [15], [16] but high cost and poor scalability limit its broader application. As a result, a sensitive and comprehensive understanding of the genetic pathways and kinetics of viral adaptation to acute phase immune selection pressures across the entire HIV-1 genome, likely a critical determinant of the success or failure of both natural and vaccine-elicited immune responses, is lacking.

Next-generation sequencing (NGS) or deep sequencing approaches such as 454 pyrosequencing [17] have the potential to transform the study of HIV-1 and other highly variable pathogens by providing a rapid and cost-effective approach for the sensitive characterization of the complex and rapidly evolving intra-patient viral quasispecies. Recent studies have applied deep sequencing approaches to HIV-1 and SIV to detect low frequency drug resistance variants [18]–[22] and CD8+ T cell escape variants [23]–[26], although these studies have largely been limited to the analysis of discrete regions of interest. Here we report an approach for routine whole genome sequencing of HIV-1 that combines deep sequencing with novel algorithms for *de novo* sequence assembly and for accurate quantification of low frequency viral variants. This new platform not only provides the capacity to rapidly sequence across the whole HIV-1 genome for population-scale genetic analyses of large cohorts of HIV-1 infected individuals, but also the sensitivity to comprehensively characterize the earliest stages of viral immune adaptation during the critical initial interactions with the host immune response. The application of this whole genome deep sequencing platform to longitudinal samples from a single subject during acute HIV-1 infection reveals the speed and complexity of the simultaneous adaptation of HIV-1 to multiple host immune responses, and suggests that early, low frequency escape variants to dominant acute-phase CD8+ T cell responses may have a significant impact on the early immune control of HIV-1.

## Results

### Whole HIV-1 genome sequencing and assembly using 454 pyrosequencing

**Figure 1:**
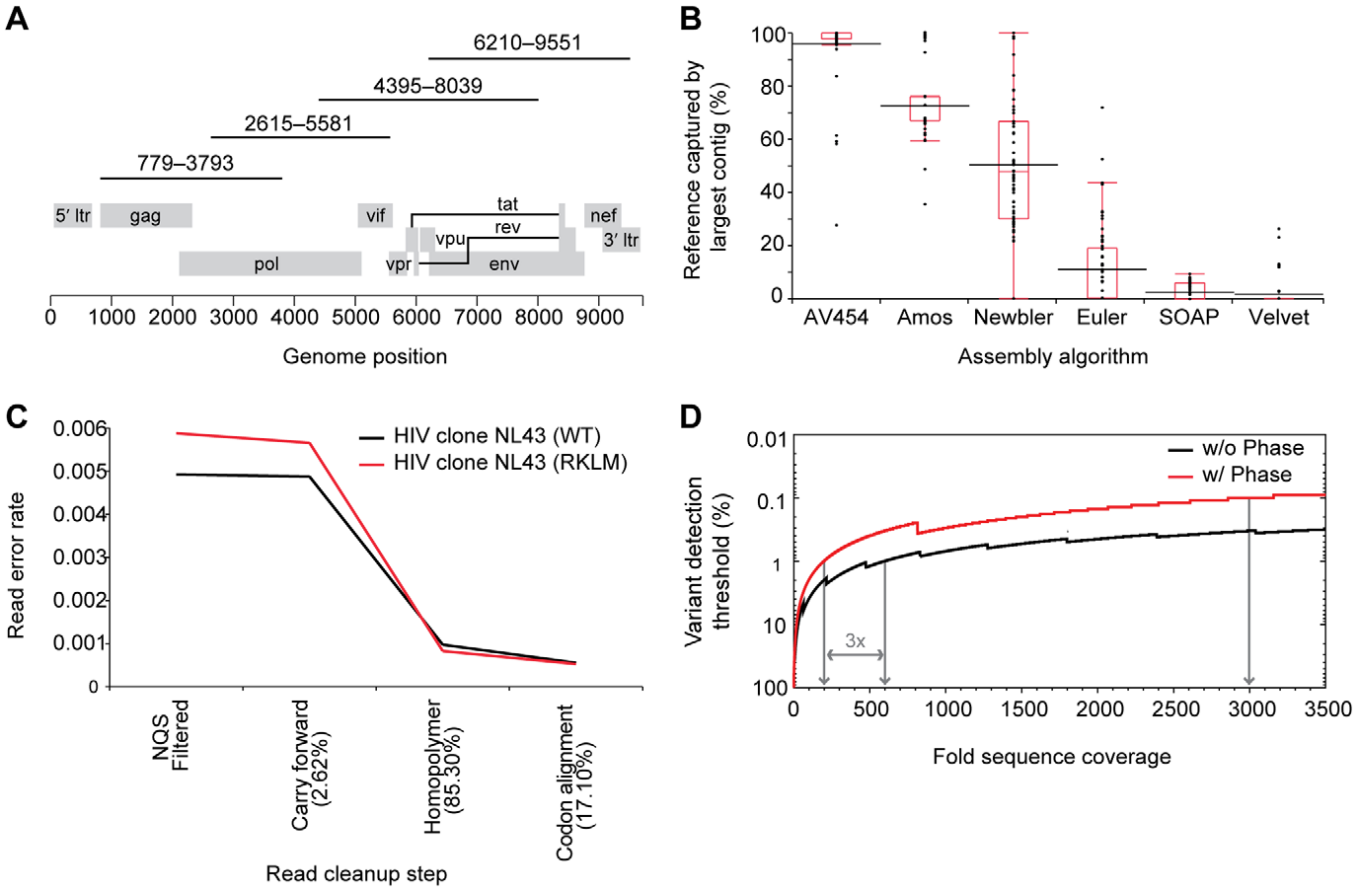
PCR amplification strategy and performance of novel assembly, read alignment, and variant detection algorithms. (**A**) PCR amplification strategy using four ∼3.2 kb amplicons spanning gag through nef of the HIV-1 genome. Amplicons were then pooled, sheared, barcoded by patient or time point, and batched for library construction and single-molecule 454 pyrosequencing. (**B**) *AssembleViral454* v1.0 outperforms other algorithms in its ability to assemble *de novo* continuous consensus contigs that span the complete target region. Results are shown for 67 acute, chronic, or controller patient samples that had successful amplification of all four amplicons and at least 10-fold sequence coverage (sequencing reads per site) across >70% of the target genome. *Black lines* denote the mean score for each assembler, *red line* the median, *red box* ends the 25th and 75th quantiles, and *red box whiskers* the upper and lower quartiles plus/minus 1.5 times the interquartile range, respectively. (**C**) *ReadClean454* v1.0 corrects for read alignment errors due to various sequence error modes and significantly reduces process error rate. Results shown are for virus from two infectious clones, NL43 (WT) and NL43 (RKLM) containing two point mutations in Gag [10], sequenced independently to 417- and 189-fold average coverage, respectively. Errors are defined as base calls or InDels that differ from the assembled consensus at a given position, and the read error rate is the total number of errors per total number of NQS passing bases interrogated. Percentage of reads on which a correction was made at each step are shown in *parentheses*. A final average process error rate of 0.5×10^−4^ was achieved based on both infectious clones. (**D**) *V-Phaser* v1.0, utilizes phasing information to identify a variant pair found in 1.0% of the reads covering both loci when there are 200 such reads; without phase, a three-fold increase in coverage is required to achieve the same 1.0% detection threshold. A variant at a frequency of 0.1% can be detected when phased coverage is 2999-fold.

Prior studies utilizing deep sequencing to more critically examine HIV-1 and SIV sequence diversity and evolution have focused predominantly on short, specific regions of the virus where evolution was known or expected to occur. To apply a deep sequencing approach that can interrogate diversity across the complete genome we designed primers that amplify four overlapping PCR amplicons spanning the entire protein-coding region of the HIV-1 genome (HXB2 nt 779–9551; **Figure 1A**) and validated them against a set of 89 HIV-1 clade B (HIV-1B) clinical samples from subjects in the acute and chronic phase of infection, as well as low-viremia controllers (**Table S1** in **Text S1**). To reduce costs, we pooled the four amplicons from each individual sample prior to acoustic shearing and subject-specific molecular bar-coding, and then batched bar-coded samples from multiple subjects prior to performing emulsion PCR and pyrosequencing.

In contrast to traditional Sanger sequence data, the 454 sequence data provides deep read coverage (sequencing reads per site) where each individual base and the context in which it occurs in the read can be leveraged to inform the consensus assembly. As such, we developed *AssembleViral454* (*AV454*), a module in the *ARACHNE* ^17,18^ assembly tool kit (see Supplementary Methods in **Text S1**), which takes advantage of deep sequence coverage and the knowledge that continuous RNA viral genomes do not generally contain large repetitive sequences to correctly assemble all reads. As shown in **Figure 1B**, *AV454* consensus assemblies captured on average 96.3%±11.3% (s.d.; n = 89) of patient-specific reads into a single contig (**Table S1** in **Text S1**), significantly outperforming the other assemblers. While both *AV454* and *Newbler* captured >98% of the target genome by all contigs assembled, *AV454* captured a significantly greater percentage of the genome in a single continuous contig than any other assembler (see Supplementary Methods in **Text S1**; Wilcoxon, p<0.001, n = 67) and exhibited a much tighter distribution of results. These data demonstrate the ability of this sequencing and assembly strategy to reproducibly generate genome-wide sequence assemblies from a wide variety of different HIV-1B clinical isolates.

### Development of novel algorithms to accurately detect low frequency variants

A major challenge to the utility of deep sequence data is distinguishing true genetic polymorphisms from process errors [22], [23], [26], [27]. We addressed this problem by developing an analysis pipeline designed to: (i) maximize the read data retained for analysis, (ii) optimize read alignments, and (iii) leverage phase information to improve the sensitivity and specificity of variant calling. First, all read alignments are made to the sample's *AV454* consensus assembly. A comparison of read alignments to the *AV454 de novo* assembly versus an HIV-1B reference sequence demonstrated that use of the *AV454* assembly retained more reads and bases for analysis and significantly reduced the number of insertions and deletions that result in alignments with frame shifts (**Table S2** in **Text S1**; Wilcoxon, p<0.0001), an important consideration for variant calling. Second, *ReadClean454* (*RC454*) applies a Neighborhood Quality Standard (NQS) base filter [28], corrects reads for common process errors such as homopolymer and carry-forward-incomplete-extension (CAFIE) miscalls (see Supplementary Methods and **Figure S1** in **Text S1**), and further optimizes read alignments using coding frame information. As shown in **Figure 1C**, *RC454* significantly reduces the average process read error rate from 1.3×10^−2^ to 0.5×10^−4^ errors per base as determined by the sequencing of infectious HIV-1 clones. Next, *V-Phaser* distinguishes true variants from sequencing errors by defining the frequency at which a nucleotide polymorphism must be observed to be considered a true variant. This is accomplished through the application of an error probability model initially defined by a uniform empirical process read error rate and then refined by the inclusion of variant nucleotide phasing information i.e. correlated sequence changes (see Supplementary Methods in **Text S1**; Macalalad et al, manuscript submitted). Lastly, *V-Profiler* calculates the frequency of each triplet codon composed of nucleotides accepted by *V-Phaser*. When applied to samples of known composition, this pipeline quantified variants with high sensitivity (100%) and specificity (97%), and implementation of the phasing-based approach achieved detection of 1.0% variants when ≥200-fold shared sequence coverage (sequencing reads per site) was attained; this represents a three-fold decrease in required coverage over non-phase based methods; **Figure 1D**; see Supplementary Methods in **Text S1**). The application of these algorithms provides the ability to rapidly characterize intra-patient HIV-1 genetic diversity, and facilitate the routine handling of deep sequencing data for whole genome assembly and variant detection, as shown in **Figure S2** in **Text S1** for all HIV-1 proteins from the array of 89 HIV-1B clinical samples.

### Benchmarking of 454 sequencing to traditional cloning and SGA

**Figure 2:**
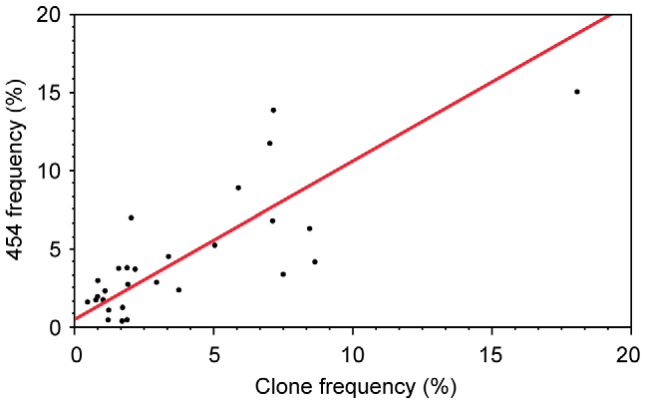
Comparison of sequence variant quantification by 454 deep sequencing and by PCR cloning/sequencing. Orthogonal regression of variant frequency estimates obtained by 454 and clonal sequence data across the highly variable 1544 nucleotide region spanning Vif to Tat in subject 9213 (slope = 1.01; 95% CI, 0.73 to 1.40).

The whole HIV-1 genome 454 deep sequence platform was validated by comparison to bulk Sanger sequencing, cloning and sequencing, and SGA. First, we compared full length consensus HIV-1 sequences for four longitudinal samples from a single subject (9213) generated by bulk Sanger sequencing and by the 454 platform (35,093 total nucleotides compared; see Supplementary Results in **Text S1**). Overall, the Sanger and 454 consensus sequences differed at only six nucleotides and one insertion/deletion (InDel), and in each case the discrepancy resulted from a differential consensus call at a highly polymorphic position (**Table S3A and S3B** in **Text S1**). Next, we extensively compared variant quantification across a highly variable 1544 nucleotide region spanning from vif to tat in a single sample (subject 9213) by deep sequencing (average 566-fold high quality sequencing reads per site), traditional PCR cloning and sequencing (768 clones), and single genome amplification (87 single genomes). We observed 95.6% concordance between the three methods in the detection of invariant/variant sites (see Supplementary Results in **Text S1**), and the calculated variant frequencies were highly correlated between methods as shown for deep sequencing vs cloning and sequencing in **Figure 2**. These data confirm the ability of this high-throughput, deep sequencing platform to profile HIV-1 quasispecies diversity as accurately as conventional cloning and sequencing or SGA.

### Characterization of whole HIV-1 genome evolution during acute infection

**Figure 3:**
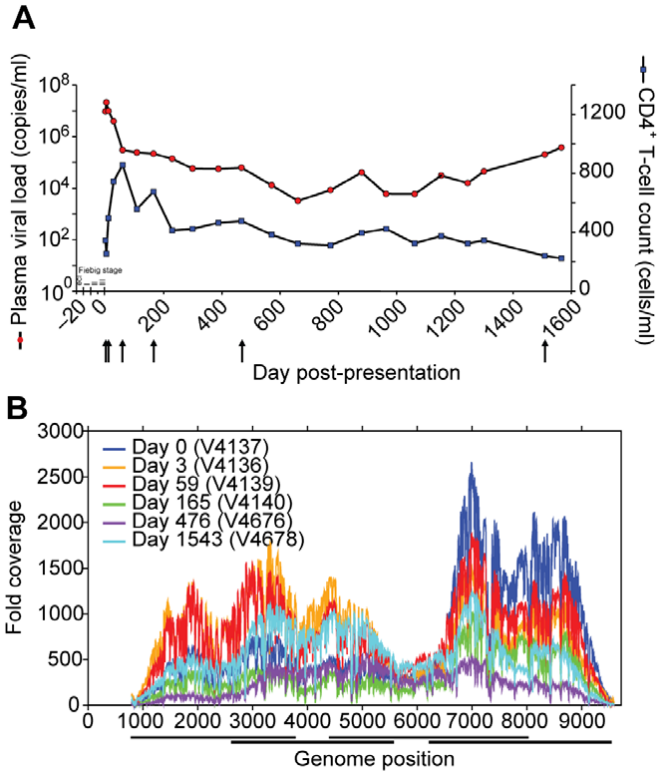
Clinical course and whole genome deep sequence coverage for subject 9213. (**A**) Clinical course of infection in subject 9213 shown as days post-presentation. Plasma viral load (copies/ml) is shown in *red* and CD4+ T cell count (cells/ml) in *blue*. Estimated acute/early Fiebig stages are shown and *arrows* indicate time points sequenced on the deep sequencing platform. (**B**) High-quality sequencing reads per site across the HIV-1 genome for subject 9213 at six time points (days post presentation). Reads are aligned to the consensus assembly of their respective time point using *Mosaik v1.0* (**Table S9** in **Text S1**) and coverage (sequencing reads per site) calculated from bases that pass the defined Neighbor Quality Standard (NQS, see Supplementary Methods in **Text S1**) [28]. PCR amplicon locations are denoted by horizontal bars under the x-axis.

**Figure 4:**
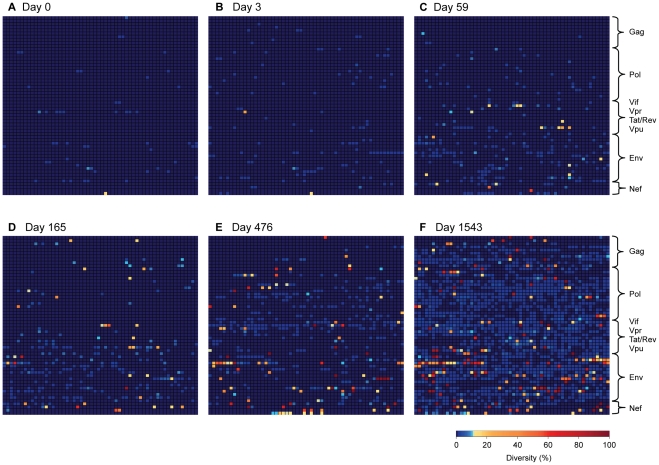
Rapidly expanding sequence diversity during HIV-1 infection. Heat maps illustrate sites exhibiting amino acid sequence diversity at days 0, 3, 59, 165, 476 and 1543 post-presentation. Plotted is the percentage of amino acid diversity at each position with respect to the dominant baseline (day 0) amino acid residue. All 3174 amino acids of HIV-1 are represented, with the first amino acid of Gag located in the top left corner of the grid and the last amino acid of Nef located in the bottom right corner. Completely conserved residues are *dark blue*, low-level variant residues (<10% divergent from baseline) are *light blue*, moderately variable residues (10–50%) in *orange*, and highly variant residues (>50%) in *red*. (**A**) 0 days p.p., (**B**) 3 days p.p., (**C**) 59 days p.p., (**D**) 165 days p.p., (**E**) 476 days p.p., (**F**) 1543 days p.p..

Recent studies utilizing deep sequencing to more sensitively assess early, low frequency variants within specific CD8 epitopes reveal that viral escape from CD8+ T cell responses can occur very rapidly [23], [25], [26], even as soon as 17 days following SIV infection of macaques [23]. To further explore the dynamics of HIV-1 evolution and immune adaptation during acute infection, we conducted a comprehensive and sensitive assessment of early viral evolution, without bias towards previously studied epitopes, by producing longitudinal genome-wide 454 sequence data from longitudinal samples from a single subject identified as HIV-1 infected prior seroconversion. Subject 9213 presented with a baseline viral load of 9.3 million copies/ml (day 0 post-presentation) that peaked at 21 million copies/ml on day 3 (**Figure 3A**). A negative Western blot on day 0 supported likely infection within 15–20 days of first sampling, i.e. Fiebig stage II–III [29]. We captured genetic diversity data for the entire open reading frame of HIV-1 at six time points over the first 4 years of infection (day 0, 3, 59, 165, 476, 1543) at an average number of sequencing reads per site of 535±325 reads (**Figure 3B** **, Table S1** in **Text S1**). Codon diversity, defined as the frequency of codons that differed from the consensus codon at baseline (day 0), was calculated for each position of the HIV-1 proteome. As illustrated in **Figure 4A and 4B**, there was strikingly little codon diversity present in the viral population during peak viremia, with less than 2% and 5% of all positions exhibiting detectable diversity at day 0 and day 3, respectively, and of those positions that did vary the majority varied by less than 2%. The low genetic diversity of the viral quasispecies during early acute infection, which would not have been discernable using traditional bulk sequencing approaches, confirms that infection in this subject was founded by a single genetic lineage, in line with recent reports suggesting that most sexually transmitted HIV-1 infections arise from a single founder virus [15], [16], [30]–[33].

### Early HIV-1 evolution is associated with immune adaptation

**Figure 5:**
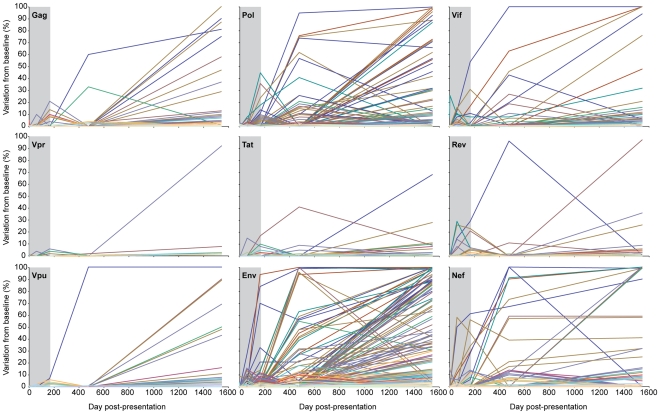
Limited evolution in the HIV-1 proteome prior to establishment of viral set point. Sequence diversity is plotted for all evolving codons in each HIV-1 protein as the percent of sequences with an amino acid residue different from the dominant baseline residue. Colored lines denote individual evolving amino acid residues within each protein. The time of infection prior to the establishment of viral set point (day 165) is highlighted in *grey*.

The first evidence of HIV-1 evolution was observed at day 59, when 11% of all codons exhibited detectable diversity (**Figure 4C**). However, still only a minor subset of 21 codons exhibited any substantial (>10%) degree of variation from baseline at this time point when peak viral loads were observed to dramatically decline to 298,000 copies/ml (**Figure 3A**). Although as expected the number of evolving codons continued to increase over time, with 38 exhibiting detectable diversity at day 165 (**Figure 4D**), it is notable that over half of the day 59 sites exhibiting substantial variation (>10%) declined in variation by day 165 (**Figure 5**). These data reveal complexities in the early evolution of the viral quasispecies that are not typically observed by traditional sequencing methods. Moreover, as shown in **Figure 5** even by day 165 no single codon had yet mutated towards fixation (>95%), suggesting that the substantial early decline in peak viremia in subject 9213 was not associated with any dramatic turnover of the viral population.

**Figure 6:**
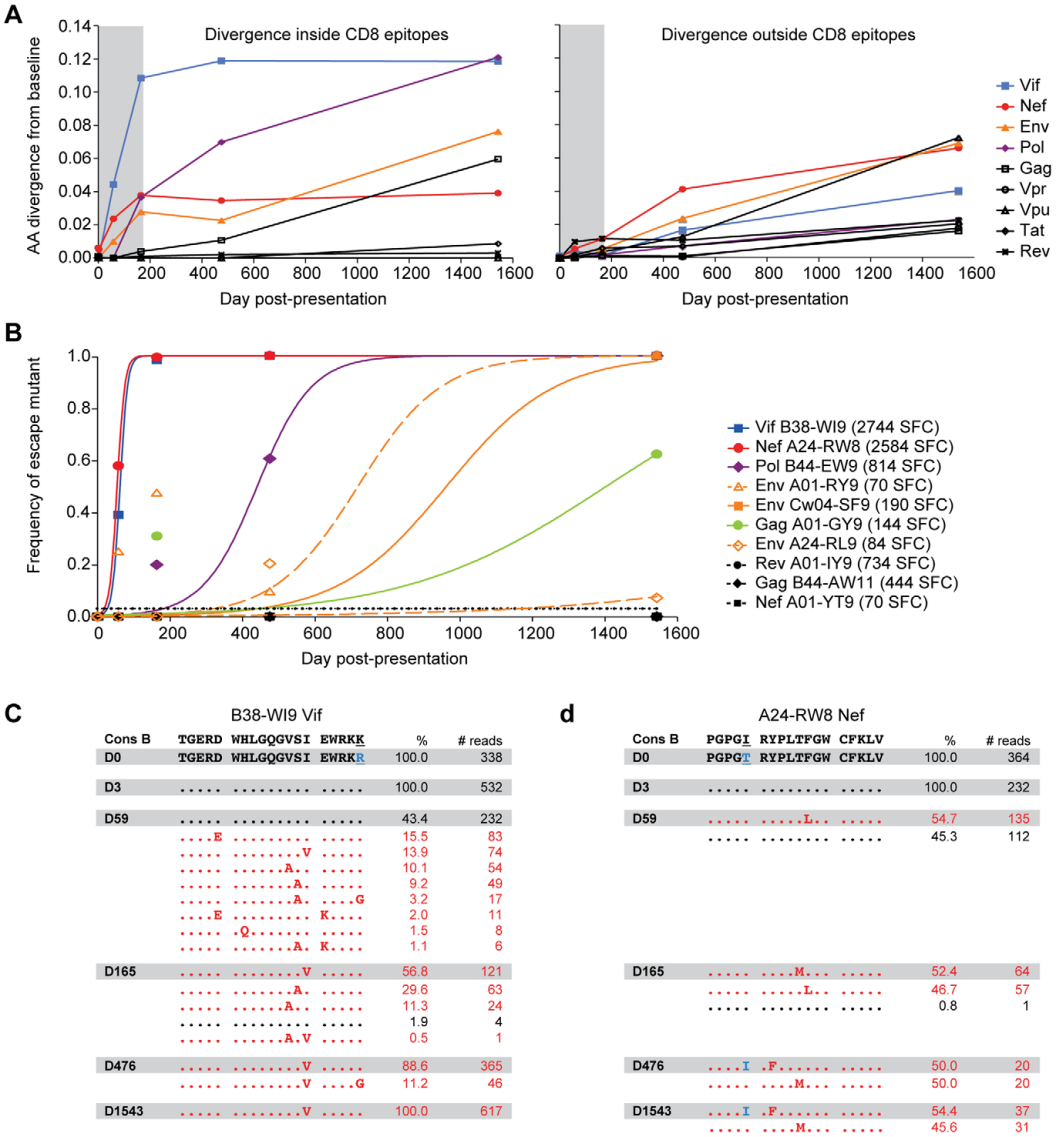
Cellular immune responses drive early low-frequency quasispecies diversity. (**A**) For each protein, the average frequency of non-dominant baseline residues of positions within the 19 described CD8 epitopes restricted by subject 9213's HLA alleles (*left*) and outside of the 19 described epitopes (*right*) is plotted for each time point sequenced. *Colored lines* denote the proteins for which diversity was substantially higher inside of CD8 epitopes versus outside CD8 epitopes. (**B**) To determine rates of viral escape for each epitope escape mutations were defined as any amino acid substitution within the epitope. *Symbols* denote the cumulative observed frequency of all escape mutations, and lines depict the best fit by non-linear regression of the observed frequency data to the CTL escape model of Asquith et al. [65]. *Open symbols* and dashed lines denote epitopes for which evolution was consistent with reversion. *Black symbols* and *dotted lines* denote epitopes for which there was no evidence of escape. CD8 responses against each epitope are shown in parentheses in the legend and were measured by IFN-gamma Elispot assay (Spot Forming Cells/Mill PBMC (SFC)). (**C**) Frequency of wild-type (*black*) and variant (*red*) haplotypes of the Vif B38-WI9 epitope and flanking regions over time. Shown at the top is the clade B consensus sequence for reference. (**D**) Frequency of wild-type (*black*) and variant (*red*) haplotypes of the Nef A24-RW8 B38-WI9 epitope and flanking regions over time. *Blue* residues highlight differences between the day 0 transmitted sequence and HIV-1B consensus sequence.

Given that CD8+ T cell responses represent a major driving force of viral evolution following acute HIV-1 infection [5], [7], [34], we examined the extent to which these early, low frequency mutations might represent viral adaptation to cellular immune responses. Here we compared amino acid divergence from baseline within described CD8+ T cell epitopes restricted by subject 9213's HLA alleles to the amino acid divergence at all other positions across the proteome. We observed that the majority of early viral evolution at days 59 and 165 was indeed shaped by cellular immune responses, with significantly greater diversity observed within restricted epitopes (Wilcoxon, p = 0.016; **Figure 6A**; **Table S4** in **Text S1)**. At day 59, this was most pronounced in Vif and Nef, with Env and Pol also exhibiting diversity preferentially within restricted CD8 epitopes by day 165. These data suggest that rapid adaptation to cellular immune responses was the major driving force for the early, low frequency viral evolution observed in subject 9213.

### Rapid viral escape from immunodominant acute phase CD8+ T cell responses

**Figure 7:**
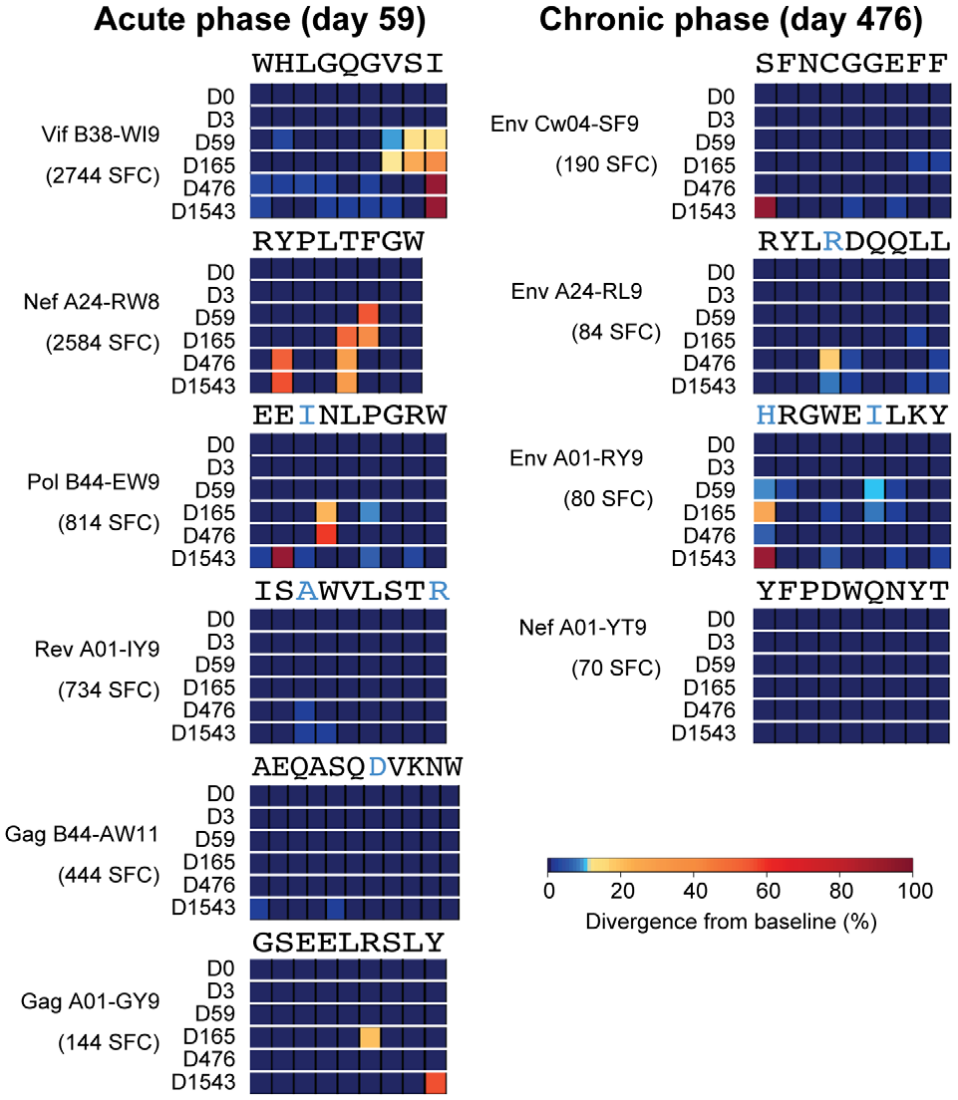
Viral escape from acute and chronic phase CD8+ T cell responses. Stacked heat-maps illustrate variant codon frequencies over time for each residue of the CD8 epitopes targeted by subject 9213. Shown are epitopes targeted during the acute (Day 59) and chronic (Day 476) phases of HIV-1 infection. The baseline sequence is shown at the top of each epitope, with non-HIV-1B consensus residues highlighted in *blue*. The magnitude of each response is shown in SFC per million PBMC.

To better understand the early immune adaptation of HIV-1 in subject 9213, we characterized the breadth and magnitude of CD8+ T cell responses to all 19 described CD8+ T cell epitopes by IFN-gamma ELISPOT assay using autologous peptides. Acute phase (day 59) responses were detected against six epitopes, with the two most dominant responses directed against the Vif B38-WI9 (2744 SFC/Mill PBMC) and Nef A24-RW8 (2584 SFC) epitopes, while weaker subdominant responses were directed against the Pol B44-EW9 (814 SFC), Rev A01-IY9 (734 SFC), Gag B44-AW11 (444 SFC), and Gag A01-GY9 (144 SFC) epitopes (**Table S5** in **Text S1**). The deep sequencing data revealed evidence of viral adaptation, i.e., escape, within four of the six epitopes (**Figure 7** **and Table S6** in **Text S1**). The escape phenotype of the observed genetic variants was confirmed by the impaired recognition of each of the variant's peptides when tested in IFN-gamma ELISPOT assays (**Table S5** in **Text S1**).

Viral immune adaptation was most rapid in the dominantly targeted Vif B38-WI9 and Nef A24-RW8 epitopes, with estimated escape rates of 0.0987 day^−1^ and 0.0976 day^−1^, respectively (**Figure 6B**). Interestingly, we observed distinct adaptive pathways by which the virus evaded each of these dominant, early responses. In the Vif B38-WI9 epitope, by day 59 56.6% of the viral population expressed one of four intra-epitope mutations (**Figure 6C**), and/or three flanking mutations likely affecting antigen processing [35], [36]. This initial apparent exploration of multiple escape pathways resolved over time with over 98% of sequenced reads from the population now comprising just three variant “haplotypes” by day 165, before fixing on the I_87_V mutation at the C-terminal HLA-class I epitope anchor residue by day 476 (**Figure 7**). In contrast, immune adaptation in the Nef A24-RW8 epitope followed a more restricted pathway with 54.7% of the quasispecies expressing a single escape mutation (F_148_L) at day 59, followed by the emergence of a second escape mutation (T_147_M) at day 165 which together comprised >99% of the total population (**Figure 6D**). Interestingly, the original F_148_L mutation at position 6 of the epitope was replaced by day 476 with the Y_144_F mutation, a position 2 HLA-anchor mutation that is likely a more potent escape mutation. This approximately 50–50 mixed population of position 2 Y_144_F and position 5 T_147_M escape variants remained stable out to day 1543 (**Figure 7**). Thus, deep sequencing during the acute phase of infection revealed rapid viral escape from the two most dominant acute phase CD8+ T cell responses, in some cases through the combined effects of multiple low frequency variants that would be missed by traditional bulk sanger sequencing. Interestingly, in both cases the early escape mutations were ultimately replaced in the viral population by HLA anchor position mutations that more efficiently escaped immune recognition (**Table S5** in **Text S1**), presumably through reductions in MHC-I:peptide binding at the cell surface.

### Slower rate of escape from subdominant CD8+ T cell responses

Viral escape was also observed in the Pol B44-EW9 and Gag A01-GY9 epitopes (**Figure 7** **and Table S6** in **Text S1**) that were targeted by subdominant acute-phase CD8+ T cell responses of 814 SFC and 144 SFC, respectively (**Table S5** in **Text S1**). Here, the lower magnitude of these responses was associated with slower estimated escape rates of 0.0133 and 0.0036 day^−1^ (**Figure 6B**). In both cases, early, low frequency mutations at positions 4 and 6 of these epitopes at day 165, likely T cell receptor (TCR) escape mutations, were subsequently replaced by HLA-anchor mutations at position 2 or 9. These data provide insight into possible mechanisms underlying the transient variation of some residues observed in **Figure 5**, whereby early mutations are being out competed by more effective secondary mutations. Finally, the two other epitopes that were targeted during acute infection, Rev A01-IY9 (734 SFC) and Gag B44-AW11 (444 SFC), exhibited no evidence of immune escape over the course of infection despite the higher sensitivity of deep sequencing (**Figure 7** **and Table S6** in **Text S1**).

In addition to the six epitopes targeted during acute infection, weak CD8+ T cell responses were also detected against four other epitopes during the chronic phase of infection (day 476): Env Cw4-SF9 (190 SFC), Env A24-RL9 (84 SFC), Env A01-RY9 (80 SFC), and Nef A01-YT9 (70 SFC) (**Table S5** in **Text S1**). There was evidence of viral escape in the three Env epitopes (**Figure 7** **and Table S6** in **Text S1**), but similar to the epitopes targeted by subdominant acute phase responses, the rate of escape in these chronically targeted epitopes was slow at 0.0067, 0.0087, and 0.0026 day^−1^ respectively (**Figure 6B**). Overall, the virus escaped from four of the six CD8+ T cell responses mounted during the acute phase of infection and three of the four CD8+ T cell responses mounted during chronic infection, with highly variable rates of escape observed for different epitopes.

### Correlation between CD8+ T cell immunodominance and rate of escape

In subject 9213, we found that the rate of immune escape from acute phase CD8+ T cell responses correlated with the magnitude of these responses (p = 0.01), reflecting the differential selective pressure imposed on the viral population by distinct CD8+ T cell responses. Interestingly, greater than 50% of the viral population had escaped the dominantly targeted Vif B38-WI9 and Nef A24-RW8 epitopes by 59 days post-presentation, which corresponds temporally to the plateauing of the precipitous decline from peak viremia and the subsequent equilibration of viral load (**Figure 3A**). Thus, these data from a single subject suggest that the rate at which the virus escapes from critical acute phase immunodominant responses, in some cases through the combined effects of multiple low frequency mutations, may influence the magnitude of the drop from peak viremia and duration of effective early immune control, and by extension set-point viral load.

### Low frequency escape mutations are associated with variant-specific CD8+ T cell responses

**Figure 8:**
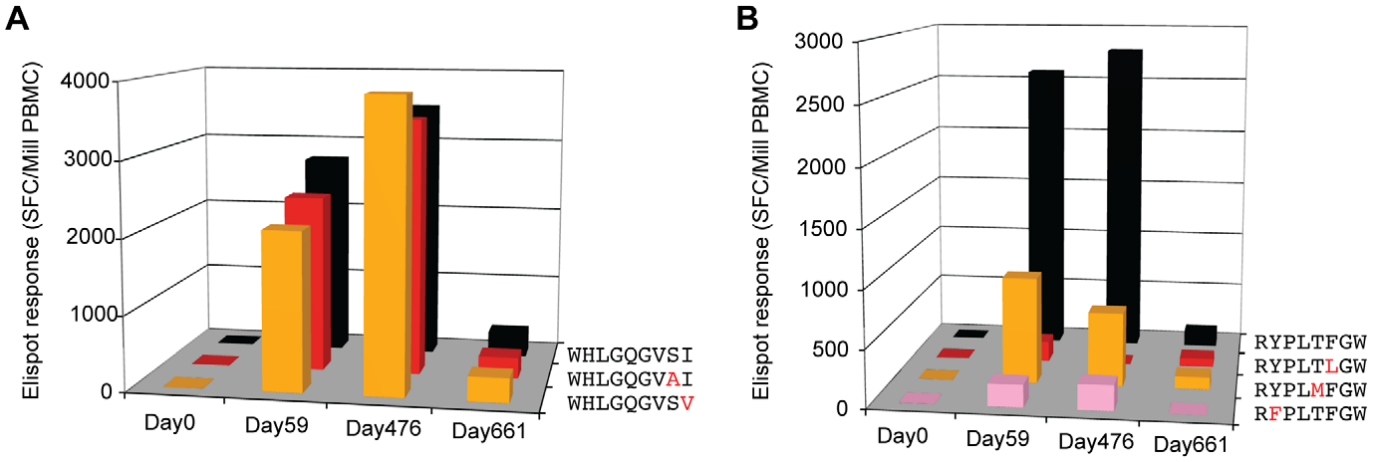
Variant-specific CD8+ T cell Elispot responses. Elispot responses in Spot Forming Cells (SFC) per million PBMC to wild-type and variant peptides for the two dominant epitopes (**A**) Vif B38-WI9 (WHLGQGVSI) and (**B**) Nef A24-RW8 (RYPLTFGW). Bars in *black* denote responses to clade B consensus epitopes. Bars in *red, orange, and pink* denote responses to epitopes containing escape variants.

We have previously observed that CD8+ T cell responses can arise that are capable of recognizing CTL escape variants [37]–[39], demonstrating that the immune system is at least partially able to contend with immune escape. To investigate the kinetics of such variant-specific responses, and whether they might be triggered by early, low frequency mutations arising during the acute phase of infection, we screened for responses against the most frequent escape variants in the rapidly escaping Vif B38-WI9 and Nef A24-RW8 epitopes. As early as day 59, strong responses were detected against two of the primary escape variants in the Vif B38-WI9 epitope, despite the fact that the S_86_A and I_87_V mutations comprised less than 15% of the viral quasispecies (**Figure 8A** **; Table S5** in **Text S1**). These variant-specific responses persisted out to day 476, and as we have previously observed in chronic infection were equal in magnitude to the autologous wild-type response [38]. However, fixation in the epitope of the C-terminal I_87_V mutation, likely impairing MHC-I binding and presentation, ultimately coincided with a significant (>10-fold) decline of both wild-type and the variant-specific responses by day 661. We also detected early responses to escape variants in the Nef A24-RW8 epitope, albeit at much lower magnitudes, and similarly the emergence of an HLA anchor position escape mutation (Y_144_F) ultimately abrogated responses against both wild-type and variant peptides (**Figure 8B**; **Table S5** in **Text S1**). This early recognition of the low-frequency escape variants, followed by loss of responses upon outgrowth of HLA anchor mutations, suggests partial cross-recognition of early escape variants by the wild-type-specific response [40] rather than development of *de novo* variant-specific responses [37]. Thus, eventual loss of the wild-type sequence, required for continuous expansion of these wild-type-specific responses, results in the eventual decline of all responses. Thus, these data extend earlier reports of the ability of CD8+ T cell responses to recognize viral escape mutations [37]–[40] by illustrating the ability of early responses to recognize low frequency escape mutations and providing a mechanism for the observed substitution of early escape mutations with more potent secondary HLA-anchor mutations.

### Reversion of transmitted escape mutations primes CD8+ T cell responses

**Figure 9:**
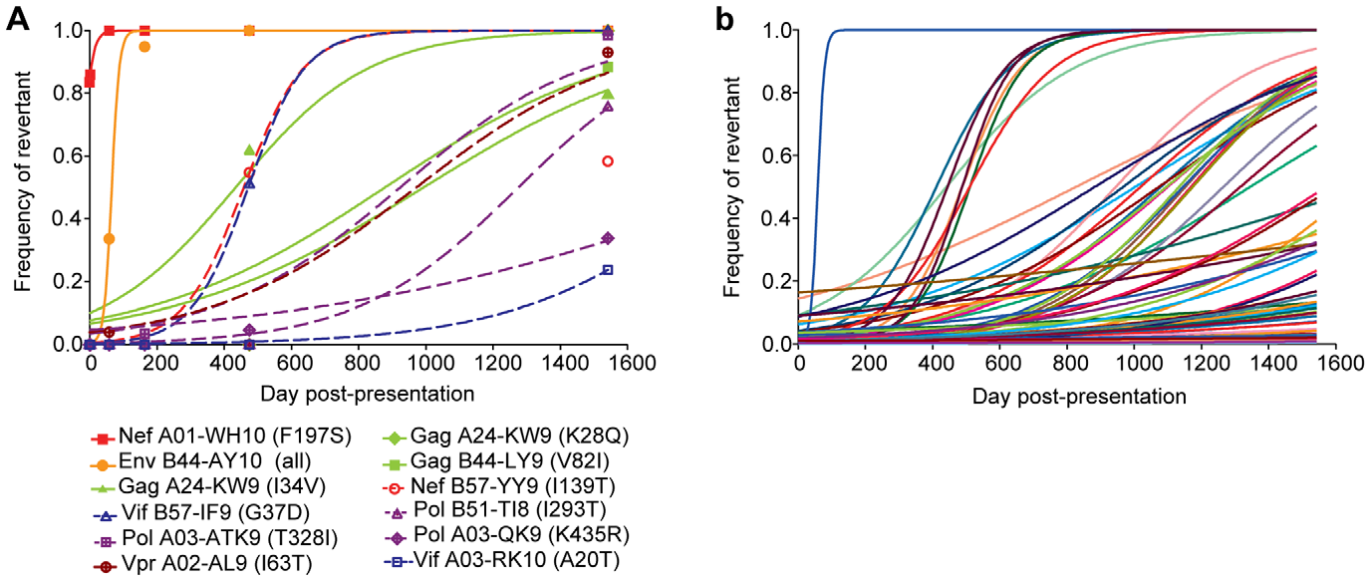
Reversion of transmitted mutations over the course of infection. (**A**) Rates of reversion of transmitted mutations within both restricted and unrestricted CD8 epitopes in subject 9213. Reversion was defined as the replacement of a transmitted non-consensus residue by the HIV-1B consensus residue. *Symbols* denote the observed frequency of viruses expressing the consensus residue and *lines* depict the best fit by non-linear regression of the observed frequency data to the CTL escape model of Asquith et al. [65]. *Closed symbols* and *solid lines* denote epitopes restricted by subject 9213, while *open symbols* and *dashed lines* denote epitopes not restricted by 9213. Listed in *parentheses* are the mutations listed by the consensus residue, HXB2 position, followed by the transmitted mutation, i.e., F197S. (**B**) Rates of reversion of all transmitted mutations exhibiting sequence variation over the course of infection. Each line represents a different mutation.

Apart from the evolution in the targeted CD8 epitopes described above, we also observed substantial evolution in four other non-targeted CD8 epitopes restricted by subject 9213's HLA alleles (Nef A01-WH10, Env B44-AY10, Gag A24-KW9, and Gag B44-LY9; **Table S7** in **Text S1**). Responses were never detected against these epitopes during either acute (day 59) or chronic (day 476 and day 661) infection despite testing with autologous peptides matching the founder virus (**Table S5** in **Text S1**). Each of these evolving epitopes was found to contain one or more transmitted mutations at baseline (day 0), with the observed evolution consistent with the reversion of these transmitted mutations back towards the HIV-1B consensus sequence. Reversions in the Nef A01-WH10 and Env B44-AY10 epitopes occurred with estimated rates of 0.0722 and 0.0887 day^−1^ (**Figure 9A**), respectively, nearly equaling those of the most rapidly escaping Vif B38-WI9 and Nef A24-RW8 epitopes (**Figure 6B**). Reversion in the Gag A24-KW9 epitope was actually the result of the transmission and reversion of a K_28_Q mutation that is a well-described escape mutation in the overlapping A03-RK9 epitope [35], [41]. Interestingly, founder virus mutations reverted in three additional HLA-A03 epitopes (Pol-ATK9, Pol-QK9, and Vif-RK10), and two HLA-B57 epitopes (Vif B57-IF9 and Nef B57-YY9), suggesting that the founder virus in subject 9213 had previously adapted to both HLA-A03 and B57 immune responses (**Figure 9A** **; Table S8** in **Text S1)**. Reversion of other well-described escape mutations such as I_293_T in the Pol B51-TI8 epitope [41], [42] and I_63_T in the Vpr A02-AL9 epitope [43] was also detected. In total, 15% (56/373) of all transmitted, non-consensus mutations exhibited evolution consistent with reversion over the four years of follow-up (**Figure 9B**). Thus, the increased sensitivity afforded by the longitudinal deep sequencing data revealed that not only is reversion of transmitted mutations a significant contributor to the evolution of HIV-1, but that these mutations revert at vastly different rates implying significantly different impacts of each mutation on viral replication capacity.

As a result of these findings we undertook a closer examination of the evolution within the ten targeted CD8 epitopes. Transmitted mutations at baseline were in fact present in five of these epitopes (**Table S6** in **Text S1**), with evolution in two of the chronically targeted epitopes consistent with the reversion of transmitted mutations. In the Env A01-RY9 epitope, despite the fact that CD8+ T cell responses were not detected until day 476, as early as day 59 low frequency mutations developed at the residues containing transmitted mutations (**Figure 7**, **Table S6** in **Text S1**). In line with the hypothesis that the early evolution in this epitope may have represented reversion of transmitted mutations, we first detected low magnitude (70 SFC) immune responses against this Env A01-RY9 epitope at day 476 following partial outgrowth of the HIV-1B consensus residue (R_794_ at 9%; **Tables S5 and S6** in **Text S1**). Similarly, in the other late-targeted Env A24-RL9 epitope we observed partial reversion (20%) towards consensus of another transmitted mutation (K_593_R) at day 476, which was also associated with the late development of a low magnitude (84 SFC) response against the wild-type form of the epitope (**Table S5 and S6** in **Text S1**). Thus, while the transmission of mutations within some CD8 epitopes restricted by subject 9213 prevented the mounting of early immune responses to these epitopes, the reversion of transmitted mutations, even at very low frequencies, was sufficient to enable the priming of immune responses to these epitopes.

## Discussion

We have established a high-throughput deep sequencing platform to assess HIV-1 sequence diversity across the entire HIV-1 genome. As the result of developing novel sequence assembly and variant detection algorithms, we were able to rapidly produce deep sequence data for a diverse set of 89 clade B clinical isolates and to dissect the evolutionary dynamics of HIV-1 during the earliest stages of acute infection. Our results from an in-depth analysis of a single subject reveal that the majority of early, low frequency mutations arising during the acute phase of infection reflect adaptation to host CD8+ T cell responses. Moreover, the temporal link observed between interruption of the decline in peak viremia and escape from the most immunodominant CD8+ T cell responses through low-frequency mutations suggests that the rate of escape from a few key acute phase CD8+ T cell responses may strongly influence primary control of HIV-1, and potentially viral set point. Thus, immune control during acute HIV-1 infection may be substantially influenced by early viral adaptations not detected by conventional sequencing approaches.

The central role of cellular immune responses in the early control of HIV-1 is highlighted by our findings that across the viral proteome the majority of early, low frequency adaptive mutations in subject 9213 were associated with CD8+ T cell responses. These data support the substantial selective pressure exerted upon HIV-1 by these responses early after infection. While limited sample availability precluded an analysis of CD4+ T cell responses, none of the rapidly evolving sites in subject 9213 arose exclusively within described CD4 T cell epitopes. While this does not exclude the possibility of CD4 escape, our data were not able to directly identify any evidence of CD4 escape. Recent studies have illustrated that HIV-specific CD8+ T cell responses are guided by distinct immunodominance hierarchies, whereby certain responses consistently arise more rapidly during the acute phase of infection, and can even dominate responses restricted by other HLA alleles [44], [45]. The two most immunodominant described B38- and A24-restricted epitopes, Vif B38-WI9 and Nef A24-RW8, were also found to be immunodominant in subject 9213. Moreover, they also represented the most rapidly escaping epitopes, with the kinetics of viral escape in subject 9213 corresponding in general to the hierarchy of all CD8+ T cell responses at baseline. These data support a strong link between the strength of a response and the relative selection pressure exerted, in line with recent data by Ferrari et al. [46]. More importantly, the observation that cessation of the rapid decline from peak viremia in subject 9213 was temporally coincident with viral escape from these two most immunodominant CD8+ T cell responses suggests that the duration of effectiveness of such immunodominant responses may be critical to the successful containment of early viral replication and prolonged viral load decline. Thus, the rate at which the earliest immunodominant CD8+ T cell responses are lost through viral escape may substantially influence the establishment of viral load set point, and thus progression to AIDS [47]. Unfortunately, with the exception of a few protective HLA alleles, the majority of immunodominant CD8 epitopes occur within more variable regions of the virus that would be expected to escape rapidly because they impart little or no viral fitness cost. As such, our data revealing that combinations of low frequency adaptive amino acid mutations may critically impact early control of HIV-1 by subverting the key CD8+ T cell responses may help to explain the inability of most HLA alleles to fully suppress early viral replication.

Characterization of the molecular pathways of viral escape is central to the rational design of a durable T-cell based vaccine. The sensitivity of our approach revealed a common pattern of evolution within the majority of escaping epitopes, including both immunodominant and subdominant responses, in which combinations of multiple low frequency escape mutations were replaced over time by HLA-anchor mutations. CD8+ T cell responses specific for the earlier escape variants were associated with selection of these “secondary” escape mutations that were substantially more effective in abrogating CTL recognition. These data, and prior reports of variant-specific responses [37]–[40], reveal the efficacy of these variant-specific responses, and suggest a potentially more important role for these responses in the control of HIV-1. It is important to note, however, that while some studies have carefully demonstrated the ability of the immune response to recognize CTL escape variants using tetramers and peptide dilutions [37]–[39], other studies have found that the high peptide concentrations often used to detect cross-reactive responses to variants can be misleading since the peptide levels are often substantially higher than physiological levels [48], [49]. Unfortunately, a lack of sample availability at the early time points when these responses were robust prevented the testing of mutant and autologous peptides at additional dilutions. Therefore, it will be important in future studies to examine the recognition of these types of early CTL escape mutations using physiological peptide concentrations of peptides, or point-mutant strains of HIV-1 so that the mutant epitopes can be naturally processed and presented at the cell surface at physiological levels. Nonetheless, these data exemplify the continuous nature of host-virus co-adaptation and suggest the need to consider these early transient escape mutations when designing vaccine immunogens. For example, mosaic immunogen approaches [50], [51], designed to impede viral escape by inducing responses against early escape mutations, may benefit from inclusion of these transient low frequency variants that are likely absent from the larger chronic sequence datasets upon which mosaic vaccine antigens are based. Similarly, these deep sequence data provide greater insight into the critical role of compensatory mutations, whereby viral escape within structurally interacting regions of a protein requires one or more co-evolving secondary mutations to retain protein structure and function [10], [52], [53]. In the Nef A24-RW8 epitope, eventual development of the position 2 HLA-anchor mutation (Y_144_F) was tightly linked to an upstream I_142_T mutation, exclusively present on the haplotype expressing the escape mutant (**Figure 6D**). Thus, supplementing existing HIV-1 sequence databases with deep sequence data from both acute and chronically infected individuals may help to identify regions of HIV-1 which require co-evolving sites to escape [54] and thus would be most susceptible to immune targeting [55], [56].

Transmitted escape mutations can also influence the course of infection both by impairing the induction of CD8+ T cell responses [35], [43], [57], but also by attenuating viral replication capacity [58], [59]. Importantly, the rate at which transmitted mutations revert may serve as a more accurate *in vivo* measurement of the relative impact of these mutations on viral fitness, as compared to *in vitro* viral fitness measurements [10]. The range of reversion rates of transmitted mutations observed in this genome-wide study (0.0887 to 0.0015 day^−1^), including some that were very rapid, supports a significant impact of some of these mutations on viral replication capacity. The ability to more accurately determine the true rates of genome-wide reversions using the more sensitive deep sequencing data provides the unique opportunity to now systematically quantify the contribution of transmitted mutations on viral fitness, which may provide additional insight into the potentially significant contribution of viral genotype to HIV-1 set-point viral load [60].

The deep sequencing approach presented here yields results consistent with those of traditional cloning or SGA. A recent study by Jordan et al illustrates similar results for sequence diversity detection between standard PCR/cloning and SGA [61]. While improving upon the sensitivity of these methods, and providing the ability to simultaneously assay genetic diversity across all residues in the genome, our variant detection methods achieve a sensitivity and specificity of >97% at a substantially reduced cost as compared to SGA or cloning. Despite this high accuracy, as with other sequencing approaches, deep sequencing has its own set of limitations. First, despite efforts to optimize read alignments, mis-alignments can occur especially at the ends of amplicons and reads and lead to false positives; *V-phaser* is designed to limit false positives and Macalalad *et al.* (manuscript submitted) have shown that the variant detection methods described here achieve a positive predicted value (PPV) of 98%. Second, 454 deep sequencing is constrained in its ability to identify long-range linked mutations beyond a single read length of approximately 400 bp. When compared to SGA, this may limit its utility to understand more complex haplotype interactions, such as whether escape mutations in two simultaneously escaping eptiopes are arising upon the same viral haplotype [7], or upon distinct viral haplotypes which later recombine [38]. Here, deep sequencing approaches and SGA may well serve to complement their respective individual strengths. Third, the bulk amplified PCR products used for this deep sequencing approach may be more prone to *in vitro* recombination events than the single-template amplifications used during SGA [62]. While this is unlikely to alter the frequency of variants detected by deep sequencing, it could limit the ability to accurately assess *in vivo* recombination rates and longer viral haplotypes. However, since both bulk amplification and SGA approaches rely on the bulk reverse transcription (RT) of RNA to cDNA, which itself may be prone to *in vitro* recombination [63], both deep sequencing and SGA approaches may still be susceptible to recombination events. Finally, given the ability to routinely sequence the viral quasispecies at near unlimited depth the issue of template resampling may be a concern. In subject 9213 we quantified the number of input RNA template molecules used for each cDNA synthesis. In each case the number of template molecules (>1000 RNA copies) was greater than the fold depth of sequence data achieved (535±325 reads), arguing against template resampling having unduly influenced our findings. Supporting this conclusion is the congruence in variants and variant frequencies observed across 454, clonal, and SGA data sets (see **Figure 2**, and Supplementary Results in **Text S1**).

The accuracy of the deep sequencing methods described here to identify variable and conserved sites are further confirmed by comparison of the diversity detected within individual patients to that observed in the global HIV-1 population. As shown in **Figure S2** in **Text S1**, which illustrates diversity plots for the 89 clade B clinical isolates, consistent diversity “hotspots” were observed in each protein, including the 5′ (p17) and 3′ (p15) regions of Gag and the V1–V3 loops of Env. Notably, sites that frequently exhibited high intra-patient diversity were more likely to be highly polymorphic in consensus sequences of circulating strains when compared both across the whole genome (Wilcoxon, p<0.0001) and within any gene (Wilcoxon, p<0.01). Conversely, 28 residues were entirely conserved in both the intra-patient and global datasets. Such data support the accuracy of the deep sequencing methods and also provide a comprehensive view of the extent of genome-wide intra-host sequence diversity achieved during chronic HIV-1 infection, revealing that sites commonly susceptible to intra-host diversity contribute directly to the diversity observed between circulating strains.

The development of a robust genome-wide HIV-1 deep sequencing approach provides both the means to rapidly produce whole genome data for large cohorts and a unique opportunity to sensitively and globally profile HIV-1's earliest adaptations to host immune pressures. Genome-wide diversity profiles may serve as a sensitive and effective readout of host immunity during both natural infection, but also following vaccination such as in the case of breakthrough subjects from the HIV-1 STEP trial [64]. Our analysis of early sequence evolution in a single subject indicates that a small number of early specific CD8+ T cell responses represent the major selective force being evaded when peak HIV-1 viremia first comes under control. Extending these results to larger cohorts of individuals, especially in subjects naturally controlling HIV-1 following acute infection, would support a critical role for the maintenance of a few key CD8+ T cell responses in the critical control of HIV-1. If so, vaccine strategies aimed at triggering immunodominant responses against critical regions of the virus may prove more effective than efforts attempting to maximize the breadth or polyfunctionality of vaccine-elicited CD8+ T cell responses [55].

## Materials and Methods

### Ethics statement

All subjects gave written informed consent and the study was approved by the Massachusetts General Hospital Review Board.

### Study subjects

Plasma samples were obtained from HIV-1 cohorts at the Massachusetts General Hospital in Boston, Massachusetts, the Jessen-Praxis in Berlin, Germany and the HIV Swiss Cohort. Subject 9213 was identified during primary HIV-1 infection (Western Blot negative; Fiebig II–III) [29], and time points are defined from day of presentation with symptomatic acute HIV-1 infection. High and intermediate-resolution HLA class I genotyping was performed by sequence-specific PCR and direct sequencing according to standard procedures.

See **Text S1** for a detailed description of sample preparation, library construction, and sequencing protocols, as well as a description of the genome assembly and variant detection algorithms and their validation.

## Supporting Information

Text S1 Supplementary document containing detailed materials and methods as well as supplementary results, tables and figures.
(DOC)
