# Supplementary material for: Whole Genome Deep Sequencing of HIV-1 Reveals the Impact of Early Minor Variants Upon Immune Recognition During Acute Infection
Source: PLoS Pathog. 2012 Mar 8;8(3):e1002529. doi: 10.1371/journal.ppat.1002529 (PMC3297584; doi:10.1371/journal.ppat.1002529)
Supplement: Text S1 — Supplementary document containing detailed materials and methods as well as supplementary results, tables and figures. (DOC) [file ppat.1002529.s001.doc]

**SUPPLEMENTARY INFORMATION**

# **Whole Genome Deep Sequencing of HIV-1 Reveals the Impact**

# **of Early Minor Variants Upon Immune Recognition During Acute Infection**

Matthew R. Henn1, Christian L. Boutwell2#, Patrick Charlebois1#, Niall J. Lennon1, Karen A. Power2, Alexander R. Macalalad1, Aaron M. Berlin1, Christine M. Malboeuf1, Elizabeth M. Ryan1, Sante Gnerre1, Michael C. Zody1, Rachel L. Erlich1, Lisa M. Green1, Andrew Berical2, Yaoyu Wang2, Monica Casali1, Hendrik Streeck2, Allyson K. Bloom2, Tim Dudek2, Damien Tully2, Ruchi Newman1, Karen L. Axten2, Adrianne D. Gladden2, Laura Battis2, Michael Kemper2, Qiandong Zeng1, Terrance P. Shea1, Sharvari Gujja1, Carmen Zedlack3, Olivier Gasser4, Christian Brander5,6, Christoph Hess4, Huldrych F. Günthard7, Zabrina L. Brumme2, Chanson J. Brumme2, Suzane Bazner8, Jenna Rychert8, Jake P. Tinsley9, Ken H. Mayer9, Eric Rosenberg8, Florencia Pereyra2, Joshua Z. Levin1, Sarah K. Young1, Heiko Jessen3, Marcus Altfeld2, Bruce W. Birren1, Bruce D. Walker2,10, Todd M. Allen2,*

1Broad Institute of MIT and Harvard, Cambridge, MA, USA; 2Ragon Institute of MGH, MIT and Harvard, Boston, MA, USA; 3HIV Clinic Praxis. Jessen, Berlin, Germany; 4Immunobiology Lab, Department of Biomedicine, University Hospital Basel, Basel, Switzerland; 5Institució Catalana de Recerca i Estudis Avançats (ICREA), Barcelona, Spain; 6Irsicaixa AIDS Research Institute-HIVACAT, Hospital University Germans Trias I Pujol, Badalona, Spain; 7Division of Infectious Diseases and Hospital Epidemiology, University Hospital Zurich, University of Zurich, Switzerland; 8Massachusetts General Hospital and Harvard Medical School, Boston, MA, USA; 9The Fenway Institute, Fenway Health, Boston MA, USA; 10Howard Hughes Medical Institute, Chevy Chase, MD, USA.

*# These authors contributed equally.*

**TABLE OF CONTENTS**

| Supplementary Methods | ……………………………………………………….. | 2 |
| --- | --- | --- |
| Supplementary Results | ……………………………………………………….. | 11 |
| Table S1 | ……………………………………………………….. | 13 |
| Table S2 | ……………………………………………………….. | 15 |
| Table S3A | ……………………………………………………….. | 15 |
| Table S3B | ……………………………………………………….. | 15 |
| Table S4 | ……………………………………………………….. | 16 |
| Table S5 | ……………………………………………………….. | 17 |
| Table S6 | ……………………………………………………….. | 19 |
| Table S7 | ……………………………………………………….. | 20 |
| Table S8 | ……………………………………………………….. | 21 |
| Table S9 | ……………………………………………………….. | 22 |
| Figure S1 | ……………………………………………………….. | 23 |
| Figure S2 | ……………………………………………………….. | 24 |
| Supplementary References | ……………………………………………………….. | 26 |

**SUPPLEMENTARY METHODS**

**Elispot Assay**

Synthesized peptides corresponding to described HLA-matched optimal and autologous epitopes were used to detect CD8+ T-cell responses from both fresh and frozen PBMC by IFN-γ Elispot assay as described previously [1]. PBMC were plated at 100,000 cells per well with peptides at a final concentration of 14 μg/ml in 96-well plates. Negative controls (media alone) were always < 30 SFC per 106 input cells and a response considered positive if > 55 SFC per 106 input cells and at least 3 times greater than mean background activity.

**Viral RNA Isolation and Quantification**

One ml of plasma was thawed at room temperature and centrifuged at 20,817 x g for 1.5 hours at 4°C after which the pellet was re-suspended in 140 µl of the supernatant. The QIAamp Viral RNA Mini Kit (Qiagen) was used to isolate viral RNA per manufacturer protocol with the exception of an additional on-column DNase treatment. Briefly, after the column was washed with Buffer AW1, 10 µl of DNase (Qiagen) diluted in 70 µl of RDD Buffer (Qiagen) was added to the column, and was incubated for 15 min. at room temperature. The column was then washed again with Buffer AW1 prior to continuing with the manufacturer protocol. Viral RNA was eluted in 60 µl of RNA Stabilization Solution (Applied Biosystems/Ambion), aliquoted, and stored at -80°C. Quantitative RT-PCR (qRT-PCR), used to assay for successful viral RNA isolation, was performed on the Stratagene MX3000p (Stratagene) using HIV-1 *gag* SK145 (AGTGGGGGGACATCAAGCAGCCATGCAAAT) and SK431 (TGCTATGTCACTTCCCCTTGGTTCTCT) primers [2] at 300 nM final concentration and the QuantiTect SYBR Green RT-PCR Kit (Qiagen) per manufacturers protocol. The quantification standards consisted of linear, near-full-length, HIV-1 clade B plasmid DNA derived from the pHXB2-RU3 plasmid [3].

**RT & PCR Amplification for 454**

For each sample, 5 µl of viral RNA and 2.5 µM oligo dT20 (Invitrogen) were incubated at 65°C for 5 min. followed by 45°C for 5 min. RT master mix (1x first strand Superscript III RT buffer, 0.5mM dNTPs, 0.01M DTT, 40U RNase OUT and 200U Superscript III RT; Invitrogen) was added to each sample and incubated for 90 min. at 45°C. An additional 200 U Superscript III RT was added to each sample and incubated for 90 min. at 45°C followed by 70°C for 15 min. After cDNA synthesis, viral RNA was removed by digestion with RNaseH (4U/sample; Invitrogen). Four overlapping PCR products of size ~3.5kb were created, using primer pairs designed by a custom-designed algorithm PRISM [4], to capture *gag* through *nef* of the HIV-1 B genome. In some cases degenerate primers were used to further reduce the influence of any potential primer binding site polymorphisms. Four primer pairs (1F:GAAGGAGAGAGAGATGGGTG; 1R:TCCCACTCAGGAATCCAGGT; 2F:ACA-ATGGCCATTGACAGAAG; 2R:TGGTCTTCTGGGGCTTGTTC; 3F:TGTAGTCCAGGA-ATATGGCA; 3R:GGTGCADATGAGTTTTCCAG; 4F:CAGAAGACAGTGGCAATGAV; 4R:ACCAGAGAGACCCAGTACAG) were synthesized with a 5’ amino modifier C6 (Integrated DNA Technologies) to prevent ligation during the 454 library construction and allow for more even sequence coverage across each amplicon [5]. PCR was performed using the high-fidelity PfuUltra II Fusion HS DNA polymerase (Stratagene/Agilent). Thermal cycling conditions were 95°C for 2 mins, followed by 40 cycles of 94°C for 30s, 62°C for 30s, 72°C for 3.5 mins, with a final extension step at 72°C for 10 mins. Resulting amplicons captured genome positions 779-3793, 2615-5581, 4395-8039, and 6210-9551 as defined by the HXB2 reference (http://www.hiv.lanl.gov/content/sequence/HIV/MAP/landmark.html). For post-PCR quality control purposes, the products were run on pre-cast 1% agarose E-Gels (Invitrogen). Each reaction was quantified using the Quant-iT PicoGreen dsDNA assay (Invitrogen). Based on these concentrations, 50 ngs of each reaction were pooled for a total of 200 ngs and the volume was brought up to 100 µL with TE for shearing and library construction.

**454 Library Construction and Sequencing of Full-Length Genomes**

Pooled PCR products were prepared for sequencing on the 454 Genome Sequencer FLX Titanium (Roche) using standard protocols with the following modifications. Samples were sheared in a 96 well plate format using an adaptive focused acoustic shear technology (Covaris, Inc. E210). Shearing conditions were as follows: time = 120 sec, duty cycle = 5, intensity = 5; cycles per burst = 500. Post-shearing, samples were size selected using a paramagnetic iron bead-based process (AMPure, Agencourt Biosciences) resulting in fragment sizes between 300 and 800 bases. Each sample received a 454 library adapter that had been synthesized with an in-house designed 5-8 base molecular barcode (i.e. multiplex identifier or MID) [6]. Post adapter ligation, batches of samples plus a NL4-3 control were pooled by volume to create a single sequence-ready library. Emulsion PCR and sequencing were performed according to manufacturer’s protocols. The libraries were loaded into one 1/4th or 1/8th region of a picotiter plate (PTP) depending on the sample batch size and a target sequencing coverage of ~250-fold for each sample. Samples requiring additional reads to increase coverage were re-pooled from the adapted fragment tubes and re-sequenced on one 1/8th region of a PTP. Sequence reads were binned by molecular barcode prior to further analysis. In the case of sample re-sequencing, a quality control step consisting of computational comparison of individual consensus assemblies and variant calls for each sequencing run is applied to ensure proper combination of cumulative sequencing data. The importance of such quality control is illustrated by the identification of a sample handling error (reverse ordering) during additional processing of 9213 day 0, 3, 59, and 165 samples. The reverse ordering error was identified and corrected by pairwise comparison of all individual sequence sets and sequence data was combined appropriately; the final cumulative 454 assemblies were confirmed by comparison to pre-existing Sanger-based assemblies. The 454 sequence read data for this study are available at the NCBI Sequence Read Archive (<http://www.ncbi.nlm.nih.gov/Traces/sra>) under study accession number SRP007924.

**Sanger Sequencing of Full-Length Genome**.

To validate the consensus assembly generated from 454 sequence data (see below) two overlapping amplicons of 5.5kb and 4.5 kb which span the full genome of HIV-1 were generated using a nested PCR protocol from viral RNA isolated from patient plasma and Sanger sequenced. The first round PCR, which generated a 9kb product, was performed with 1x High Fidelity Platinum PCR buffer, 3mM MgSO4, 2 mM each dNTP, 0.5 uM each primer (R1For1 5’ AAA TCT CTA GCA GTG GCG CCC GAA CAG 3’ and R1Rev1 5’ ACG TGC CCT CAA GGC AAG CTT TAT TGA GGC 3’) [7], and 2.5 Units High Fidelity Platinum Taq polymerase (Invitrogen) in a 50 µL reaction. The reaction conditions were as follows: 94°C for 1 minute, 65°C for 20 sec., 68°C for 10 min., 14 cycles of 94°C for 15 sec., 65°C for 20 sec., 68°C for 10 min., then 20 cycles of 94°C for 15 sec., 65°C for 20 sec., 68°C for 10 min. + 20 sec. each additional cycle, and a 20 minute extension at 68°C. Samples were stored at -20°C. The second round of PCR was performed with different primer pairs (R2For1 5’ CAG GAC TCG GCT TGC TGA AGC 3’ and R2Rev1 5’ CCC TAG TGG GAT GTG TAC TTC TGA AC 3’; R2For2 5’ TCT GGA AAG GTG AAG GGG CAG TAG 3’ and R2Rev2 5’ GGT CTA ACC AGA GAG ACC CAG TAC AG 3’) [8,9]. Reaction conditions were as follows: 94°C for 1 minute, 65°C for 20 sec., 68°C for 4.5 min., 14 cycles of 94°C for 15 sec., 65°C for 20 sec., 68°C for 4.5 min., then 20 cycles of 94°C for 15 sec., 65°C for 20 sec., 68°C for 4.5 min. + 5 sec. each additional cycle, and a 20 minute extension at 68°C. Samples were stored at -20°C.

Positive PCR products were purified using the QiaQuick PCR Purification Kit (Qiagen) and concentrations were determined using the Thermo Scientific Nanodrop 1000 spectrophotometer (Nanodrop Products). A set of 70 primers based on HIV-1 Clade B sequence data were then used to generate bidirectional overlapping full genome sequence data as previously described [10]. Sequencing reactions were performed using the Big Dye Terminator 3.1 sequencing kit following manufacturer’s protocol (Applied Biosystems). Sequencing reactions were purified using DTR Ultra purification plates (Edge Biosystems) following the manufacturer’s suggested protocol. The purified sequencing reaction was run on an ABI3130 Prism automated sequencer using the RapidSeq36_POP7_1 Run Module. Ab1 files were analyzed using *Sequencher* *v4.8* (GeneCodes Corp. Ann Arbor, MI). After sequences were manually cleaned and edited for mixed bases, they were aligned by protein using *MacVector* *v11.0* (MacVector). Sanger sequencing derived consensus assemblies from this study are available at the NCBI (http://www.ncbi.nlm.nih.gov) under accession numbers JQ416158-59, JQ416161-62.

**Clonal Amplification and Sanger Sequencing of Vif**

The 2615-5581 PCR amplicon was reamplified using the original primers for 20 cycles using the high fidelity enzyme Phusion (New England Biolabs Inc.). A 1.5kb fragment of this product was digested by XbaI-SacI and then cloned into the pCR2.1 vector using a TOPO TA cloning kit (Invitrogen). Five microliters of the cloning reaction was used to transform 50 µL of Top10 strain (Invitrogen) chemically competent cells, and transformed cells were grown in SOC media for 1 hour at 37°C before plating onto agar plates containing 25 mg/ml Ampicillin and IPTG/X-Gal. The plates were grown for 16 hours at 37°C and colonies picked into a 384-well plate containing 25 mg/ml Ampicillin and 10% glycerol. The glycerol plates were grown overnight at 37°C before proceeding to a standard alkaline lysis DNA prep. Plasmid DNA was prepared by standard protocols, and cloned inserts were bidirectionally sequenced with M13 primers using the BigDye Terminator v3.1 chemistry (Applied Biosystems). Sequencing reactions were analyzed on an ABI 3730xl capillary electrophoresis sequencer (Applied Biosystems). The clone sequence read data from this study are available at the NCBI trace archive (http://www.ncbi.nlm.nih.gov/Traces/home/) under trace identifiers 2330536479-2330538014.

**Single Genome Amplification (SGA) and Sequencing of Vif**

cDNA was serially diluted and amplified in replicate in 96-well plates to identify the dilution yielding PCR success rates of <30% at which point the majority of amplicons are derived from a single copy template as previously described [11]. PCR was performed with 1x High Fidelity Platinum PCR buffer, 2mM MgSO4, 0.2 mM each dNTP, 0.2 uM each primer, and 0.025 units/uL High Fidelity Platinum Taq polymerase (Invitrogen) in a 20 µL reaction. First round PCR was performed with 1uL of cDNA, forward primer F3fF 5’-CTATGTAGA-TGGRGCAGC-3’, and reverse primer F5fgR 5’- GTCTGTGGGTACACARGC-3’ [10]. Thermal cycling conditions were 94°C for 2 min, followed by 35 cycles of 94°C for 15s, 55°C for 30s, 68°C for 4 min, with a final extension step at 68°C for 20 min. 2uL of first round PCR product was used for the second round with forward primer F3gF 5’-GGTACCAGCACACAAAGG-3’ and reverse primer F5eR 5’-ATTGCCACTGTCTTCTGC-3’ [10]. Thermal cycling conditions for the second round included 45 cycles but were otherwise the same. The presence of amplified product was tested on a 2% agarose gel. Products from dilutions that yielded <30% positive wells were purified (PCR purification kit, Qiagen) and sequenced. Bidirectional sequencing was carried out with the BigDye Terminator v3.1 Cycle Sequencing Kit (Applied Biosystems) according to manufacturer instructions, and reaction products were analyzed on an ABI 3130 Prism automated sequencer. The following primers were used to provide sequence coverage of the *vif* region: F4eF 5’-GTGTGGCARGTA-GACAGG-3’, F4eR 5’-TCTTCYGGGGCTTGTTCC-3’, F5abF 5’-TGGAAAACAGATGGC-AGG-3’, F5aR 5’-CTCCCTGRCCYARATGCC-3’, F5bR 5’-TCTTCYGGGGCTTGTTCC-3’, F5cF 5’-ACCCYGRMCTAGCAGACC-3’, and F5cR 5’-GACTTCCTGGATGCTTCC-3’12. Sequences were edited and aligned with Sequencher (Gene Codes Corp.). Sequences with mixed bases were discarded from analysis. The SGA sequence read data from this study are available at the NCBI ([http://www.ncbi.nlm.nih.gov](http://www.ncbi.nlm.nih.gov/)) under accession numbers HQ642975-HQ643081.

**454 *de novo* Consensus Assembly of Full-Length Genomes**

We developed an in-house assembler *AssembleViral454 v1.0* (*AV454*) capable of generating contiguous HIV-1 consensus assemblies from ultra-deep 454 read data sets and compared *AV454* to both De Bruijn Graph (Euler [12], Newbler (454 Life Sciences), Velvet [13], and SOAP [14]) and reference-based (AMOS [15]) assemblers. *AV454* was run as described below. All other assemblers were run as follows: (i) *Euler* *vSR.1.1.2*, K=25, default all other options; (ii) *Newbler* –ace, -ar, -nrm, -rip, -scaffold options invoked; (iii) *Velvet v0.7.47*, K=25, -exp_cov option invoked; (iii) *SOAP* *v1.03*, K=23, default all other options; (iv) *AMOScmp* *v2.0.2*, default options. Performance of the assemblers was evaluated based on the percent of the sequenced region captured by the largest continuous contig (**Figure 1B**, **Table S1 in Text S1**). *AV454* is a specialized version of the *ARACHNE* [16,17,18] assembly algorithm suitable for *de novo* consensus assembly of highly diverse 454 reads. This version of the algorithm takes advantage of deep sequence coverage as well as knowledge that in general RNA viral genomes do not contain repetitive sequences of significant size and that they are continuous genomes. The algorithm assumes that no sequence repeat would be large enough to fully contain an average read and it relaxes the dependence on read pairing since these data are often not available in 454 read data sets. The assembly process consists of two steps. First, an initial pre-processing stage is run where reads are clustered into small overlapping contigs. This is identical to the process employed in the published *ARACHNE* [16,17] algorithms with the exception that lenient alignment parameters are utilized that allow for very short overlaps to be valid; this modification takes into account that some regions of the genome will consist of highly diverse reads given the quasispecies. The output of this first step is typically a fragmented consensus assembly that is broken in some regions of the genome where read variability is high due to the viral quasispecies. Second, we employ an iterative procedure that incrementally merges the fragmented assembly that results from step one and improves read placement. This procedure works by first aggressively merging the existing contigs based on the above assumptions. Following this merging process, low quality bases are removed from the ends of the resulting contigs (bases are removed up to the point in the contig where there are 12 or more consecutive bases having quality scores of 40 or better), and regions are identified where assembly errors are likely (small repeat units that may be misassembled and regions where coverage drops below 10% of the average assembly coverage). The algorithm then removes reads falling into these potential error regions and attempts to relocate these reads to better placements in the improved assembly. Reads that can be aligned uniquely and with a good alignment score (as defined in the standard *ARACHNE* assembler) are 're-placed' in the assembly. This process then repeats until no further merging, or improved read placements are possible.

Resulting *AV454* assemblies were checked for accuracy by identifying loci with InDels that cause coding frame-shifts. Assemblies were evaluated using a two-step process. First, GeneWise [19] was used to align the consensus assembly to the protein sequence of a reference HIV-1 annotation, and these alignments were then parsed to identify InDels that break frame. The HIV-1 reference annotation was a consensus sequence generated from all available complete, non-recombinant HIV-1 clade B genomes available through the Los Alamos HIV database. Second, loci in the genome flagged as InDels were manually inspected to determine if underlying reads supported the InDel or the InDel was in a homopolymer region. Assemblies were corrected as appropriate.

Final assembly and annotations based on 454 data are available at the NCBI (http://www.ncbi.nlm.nih.gov) under accession numbers JQ403019-JQ403107. AV454 software and other project information can be obtained at the Broad Institute’s Viral Genomics Initiative homepage (http://www.broadinstitute.org/scientific-community/science/projects/viral-genomics-initiative).

**High-Sensitivity Population Profiling of Quasispecies Variants**

We mapped nucleotide and amino acid frequencies across the complete consensus assembly using a multistep process that first aligns the reads to a consensus assembly, then improves the alignments based on sequence dependent issues (*ReadClean454 v1.0* abbreviated *RC454*), and lastly identifies true variants from sequencing errors based on a defined probability of the variant being an error given an empirically determined process error rate (*V-Phaser v1.0*). The first step of this process utilizes *Mosaik* *v1.0.1388* (<http://bioinformatics.bc.edu/marthlab/Mosaik>) to align all reads to the consensus assembly generated from the same read set. Parameter settings for the *Mosaik* alignment can be found in **Table S9** in **Text S1**. Following this initial alignment, modifications are made to the reads to account for Carry Forward and Incomplete Extension (CAFIE) errors inherent in 454 data [20]. These sequencing errors occur when a homopolymer stretch of nucleotides is under-called by the base calling software and the remaining base appears in the next flow run on the sequencer. This issue creates a characteristic pattern in the nucleic acid sequence of the read whereby the last base of a homopolymer region is observed one flow cycle from the homopolymer region and this base is in disagreement with the reference assembly (**Figure S1** in **Text S1**). We correct this base-calling error by moving the incorrectly placed base to the end of the homopolymer run unless the same pattern is found in 25% or more of the reads; in which case we assume the discrepancy is a true variant. Following the correction of reads for CAFIE errors the reads are re-aligned to the reference assembly using *Mosaik* (see **Table S9** in **Text S1** for parameter settings). In the second step of this process, 454 base-calling errors (i.e. undercalls and overcalls) in homopolymer regions are corrected based on the new read to reference alignments (see **Figure S1** in **Text S1**). In the case of an under call (i.e deletion in the read), an N base with a phred quality score (Qscore) of 1 is added in the read to complete the homopolymer. In the case of an overcall (i.e. insertion in the read), the homopolymer bases with the lowest Qscore are deleted in order to correct the length of the homopolymer based on the reference sequence. Following the correction of reads for homopolymer base-calling errors the reads are re-aligned to the reference assembly using *Mosaik* (see **Table S9** in **Text S1** for parameter settings). In the third step of this process, InDels in non-homopolymer regions are evaluated to determine if they cause frame-shifts in the coding sequence. InDels that cannot be regrouped in multiple of 3s within a window of 21 bases (10 bases on each side of the central gap) are corrected. Like CAFIE errors, the correction only happens if less than 25% of the reads contain this specific sequence. Following this final read correction reads are re-aligned to the reference using *Mosaik* (see **Table S9** in **Text S1** for parameter settings).

To determine nucleotide frequencies at each locus, we applied a Binomial model that incorporates phase information (i.e. correlated change) to identify variants from sequencing errors prior to calling frequencies. First, we evaluated each locus to identify variants that occurred too frequently among the reads to have arisen entirely from error under our model. We divided the genome into homopolymer and non-homopolymer regions, and we made the assumption that these regions had uniform but distinct error rates. We determined error rates empirically by calculating mismatch rates in infectious clones sequenced in parallel with our samples of interest. We then used these error rates to estimate Px(k), the probability that k or more errors occurred at position x:

(1)

where Dx is the depth of coverage at position x, Kx is the number of errors at position x, and p is the probability that an error occurred at any one base. We recognized variants when they met or exceeded the threshold kx:

(2)

where α is the significance level, and c is the total number of positions used to apply the Bonferroni correction. This initial step is analogous to that described by Wang *et al.* [21].

Next, we used the model to find variant pairs correlated by linkage disequilibrium; this is valid under our model assumption that sequencing errors are uniformly distributed and uncorrelated. We defined Pxy(k) using the multinomial probability mass function to be the probability that errors occurred at both position x and position y on the same read for k or more reads:

(3)

where Dxy is the shared depth of coverage of position x and position y, Ixy is the number of reads with an error at position x but not position y, Jxy is the number of reads with an error at position y but not position x, Kxy is the number of reads with errors at both position x and position y, p is the probability that an error occurred at any one base, and q is 1 – p. We capture correlated variants that occurred at least as frequently as the threshold kxy:

(4)

where α is the significance level, and b is the total number of position pairs x and y that are found to occur on at least one shared read.

To call final codon frequencies, we constructed a sensitivity table based on the above defined Binomial model that indicates the number of times a nucleotide variant needs to be seen at a given sequence coverage, in-phase or without-phase, to be considered a true variant at a defined sensitivity. A list of accepted nucleotides is determined at each locus from the bases that are observed a number of times equal to or exceeding that required by the sensitivity model and actual sequence coverage at the specific locus. *V-Profiler* then calculates codon frequencies for each triplet composed of three accepted nucleotides. In all cases, only bases that pass the specified Neighborhood Quality Standard [22] (NQS) are counted; to be considered, a base must have a quality score of ≥Q20 and occur in a neighborhood of flanking bases (i.e. ±5 bases) with a quality score of ≥15. Variants observed at the ends of reads in primer binding sites were not filtered from the final variant analysis. As such low frequency variants may be detected in these regions that result from poorly-aligned, untrimmed primer sequence. Such variants do would represent true biological variants. All variants observed in these regions in our data set were non-functional and do not impact escape analyses.

*V-Phaser* does not explicitly account for the possible presence of chimeric reads formed during the PCR process, but we expect such events to impact <2% of all reads as demonstrated by Zagordi et al [23]. Macalalad et al. (manuscript submitted) discuss the impact of chimeric reads on *V-Phaser* variant calls.

For comparison of variant calls across samples from multiple time points, consensus assemblies are aligned and genome coordinates are based on the alignment.

*RC454*, *V-Phaser*, and *V-Profiler* software can be obtained at the Broad Institute’s Viral Genomics Initiative homepage (http://www.broadinstitute.org/scientific-community/science/projects/viral-genomics-initiative).

**Impact of Reference Sequence on Alignment Quality**

To determine if the reference consensus assembly used for read alignment impacted the amount of data retained for analysis or the quality of the alignment, we utilized all samples where the assembly covered 90% or more of our target region (73 samples) and then aligned the reads either to a *de novo* *AV454* assembly or to an HXB2 reference sequence. The reference sequence in this case is a modified HXB2, where the frame-shift in Vpr was fixed and the sequence trimmed to match our targeted region (779-9551 of the original HXB2). The reads were cleaned up with *RC454* and the resulting alignments were analyzed to compare the percentage of reads and bases that aligned as well as to determine the percentage of gaps in the aligned reads that were not a multiple of three (i.e. gaps that result in a frame shift and are more likely due to length polymorphisms).

**Haplotype Reconstruction and Frequencies Across Epitopes**

To determine the frequencies of haplotypes in epitope regions, we employed a three-step process. First, we selected all reads that completely span the epitope of interest. Second, we define a list of accepted haplotypes. For each read we determined the codon sequence across the epitope region of the read and scored each codon as accepted or rejected using the same criteria defined above for codon frequency calling. A haplotype sequence is defined if the entire sequence of codons overlapping the epitope of interest is comprised of accepted codons. Lastly, haplotype frequencies for all epitope regions are determined by counting the occurrence of each haplotype on the accepted haplotype list for a given epitope. Note that during this final step a read is counted regardless of its base qualities as long as it contains a complete haplotype sequence that was defined during the reconstruction step as accepted based on the codon frequency model.

**Epitope Diversity Analysis**

To evaluate the extent of viral genetic diversity observed within versus outside of regions in the genome coding for epitopes recognized by the study subject, we calculated a divergence score for each position in the genome and then compared these scores between epitope-coding and non-epitope-coding regions of the HIV genome. Genetic divergence from baseline (i.e. day 0) was determined for each amino acid position by calculating the percentage of amino acids in a given position that were different from the primary amino acid found at baseline. After obtaining a per residue divergence score three separate comparisons were made. First, we determined whether the level of divergence observed at individual residues was significantly different between residues found within epitopes versus outside of epitopes within the same gene. Second, we identified specific epitopes within a given gene that showed a significantly different level of divergence as compared to background non-epitope regions in the same gene. Lastly, we determined at the global scale (i.e. across the whole genome) whether the level of divergence observed in epitopes exhibited significantly different levels of divergence than non-epitope-coding regions.

For the first analysis, the distribution of divergence scores for residues in epitope-coding regions was compared using a one-way Wilcoxon test to a background distribution of divergence scores for residues falling outside of epitope-coding regions in the same gene; each gene was evaluated separately.

In the second analysis, a divergence score for each epitope was calculated by summing the divergence scores of each residue in the epitope. We then determined whether the divergence score for the individual epitope fell within the top 5th-percentile of divergence scores calculated for non-epitope-coding regions in the same gene (i.e. background divergence; p-value = 1-percentile). The background distribution used in this comparison was calculated using a sliding window strategy with a window size of nine amino acids and a step of one. Divergence scores were calculated as they were for the epitope for all windows that did not overlap, fully or in part, with any epitope-coding region within the gene of interest or an epitope-coding region in an overlapping gene if such existed. A window size of nine was chosen as it reflected the average length of the epitopes recognized by the study patient.

In the third global analysis, both individual epitope and individual background window divergence scores were calculated as in the second analysis. In this global analysis, two comparisons were made using a one-way Wilcoxon test. For epitopes falling in regions of the gene that do not overlap other genes, the distribution of epitope divergence scores was compared to the background distribution derived from regions of the genome that did not have overlapping genes. Epitopes falling in regions where there are gene overlaps were compared to a background divergence scores derived from gene overlap regions.

**Intra- and Inter-Host Diversity Hotspot Comparison**

We identified regions of the genome that consistently demonstrated high levels of diversity in chronic intra-host samples (i.e. hotspots) and determined whether these residues were also highly diverse in the circulating global population of HIV-1b sequences. We identified diversity hotspots across the intra-host samples by first determining the number of samples that exhibited detectable intra-host diversity (>0%) at a given residue. Following alignment of the consensus sequences for each sample, we calculated for each residue a sum score (i.e. number of genomes with diversity), and then conducted a permutation test (repetitions = 1000) keeping the maximum sum score across all residues for each repetition. We compiled a ranked distribution of max sum scores, and residues that had a sum score ≥95th percentile where flagged as highly diverse sites.

We next determined whether sites identified as highly diverse across the intra-host samples were more likely to show a high level of diversity in the circulating global population (i.e. inter-host diversity). We first obtained curated alignments for HIV-1b for each gene from the LANL HIV Sequence Database (www.hiv.lanl.gov; 2009 sequences, recombinants excluded). These alignments contained 533, 1383, 1039, 687, 341, 344, 561, 445, and 646 sequences for Env, Gag, Nef, Pol, Rev, Tat, Vif, Vpr, and Vpu respectively. We profile aligned the LANL alignment to our intra-host alignment and then mapped high diversity flags determined using the permutation test described above to the LANL alignment. We used a Wilcoxon Test to determine if sites found to be highly diverse in the intra-host samples were more likely to be highly diverse in the global population. We also identified residues that were 100% conserved in both datasets.

**Quantification of Escape and Reversion Rates**

Rates of escape and reversion were estimated from observed longitudinal mutation frequency data according to the model of escape kinetics as described by Asquith *et al.* [24]. Briefly, if *x(t)* and *y(t)* are the frequencies of mutant and wild type viruses at time *t*􏰈respectively, then *p(t)*, the proportion of viruses expressing the mutation at time (*t*), is


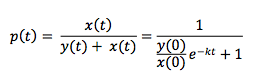


where *k* is the rate of change. The value of *k* was estimated by fitting the model to the observed data using the Levenberg-Marquardt method of non-linear least squares regression in *SigmaPlot v11.0* (Systat). For epitopes that lacked multiple data points with both wild type and mutant viruses present, the rate was estimated by substituting the observed mutant frequency of 0 or 1 with 1 ⁄ (*n*+1)􏰅or *n* ⁄ (*n*+1) respectively where *n* is the number of sequences analyzed as previously described by Goonetilleke *et al.* [25].

**Validation of assembly and variant calling derived from 454 data**

Consensus assemblies generated from 454 data by *AV454* were compared to consensus assemblies generated from traditional Sanger sequence and assembled by *Sequencher v4.8*. For patient subject 9213, we aligned the 454 consensus assembly for each time point (days 0, 3, 59, and 165) to its respective Sanger assembly using BLASTN [26] and catalogued the differences observed in the consensus call. When differences in the consensus base call at a position were detected the 454 data was evaluated to determine if the variant allele was supported by the ultra-deep 454 read data.

To test the capability of 454 sequencing to quantitatively capture intrahost diversity at a single time point, we generated diversity data by two alternate methods: sequencing of cloned PCR products (TOPO-Clone) and single genome amplification (SGA) followed by direct sequencing of PCR products (see data generation methods above). 454 data were processed as above described. TOPO-clone PCR products were sequenced from both ends and then forward and reverse reads were aligned to the reference and merged to form single composite sequences (to avoid double counting bases called on both forward and reverse). Bases seen in both forward and reverse were assigned the sum of their quality scores if the calls agreed. Discrepant bases were resolved based on quality score and assigned the difference of the quality scores. Bases that were not cleanly aligned and gaps between forward and reverse reads were assigned N for alignment and a quality of zero and were not used in analysis. Forward or reverse reads whose mate failed to align with reasonable quality to the reference were retained as single reads in the analysis. SGA sequences were manually assembled and reviewed; all assembled bases were considered high quality for purposes of variant calling.

We aligned 454, clone, and SGA reads to the reference genome and called variant codon frequencies using the variant calling pipeline above described, including phasing information on all three data types. For 454 variant calling, we used our standard empirical base quality method calibrated on 454 sequences from an infectious clone HIV-1 sample. For the clone and SGA sequences, we used PCR data from an infectious clone sample of Dengue to calibrate the sensitivity model. At least two observations of a variant base were required to call a variant, even from SGA.

454 and TOPO-clone variant frequencies were compared by orthogonal regression using *JMP v8.0.1* statistical software (SAS Inc.) with confidence intervals of the slope calculated as described by Tan & Iglewicz [27]. Sites shared between SGA and the two other methods had too few data points to generate meaningful correlations or regressions. In addition, for all sites called by only a subset of methods, we computed a hypergeometric probability of having observed the number of reads seen or fewer using the method/s which missed the call.

**SUPPLEMENTARY RESULTS**

**Comparison of AssembleViral454 Consensus Assembly to Traditional Assembly**

Alignment of each Sanger consensus assembly to its complement *AV454* consensus indicated that the two approaches yielded highly congruent results; in total six substitutions and one indel were identified. In two of the comparisons we detected no differences and in a third only a single difference was observed (**Table S2A** in **Text S1)**. In the later time point, we observed five substitution differences between matching assemblies and a 12 bp indel (**Table S2A** in **Text S1**).

To determine whether these six differences resulted from sequencing or assembly error or represented underlying variation in the samples, we looked at the estimated allele frequencies at each position showing a difference at any time point. Coordinates presented are on the 454 consensus for day 0, but we note that all four 454 time point assemblies have identical coordinates except for the day 165 assembly which has 12 deleted bases relative to the baseline. For each of the discrepancies we observed underlying variation in the sample at high enough levels to explain the differences in consensus calling (**Table S2B** in **Text S1**).

**Validation of Population Profiles by Cloning and SGA**

Sequence data from TOPO-clone and SGA overlapped the *AV454* genome at 1544 positions, spanning all of the Vif and Vpr genes and parts of Pol and Tat (positions 3670 to 5213 in our 9213 day 0 454 reference sequence). Average sequence coverage of this region by 454 was 566x high quality bases, with a maximum of 1127. We generated 768 cloned PCR products, for an average coverage of 373 high quality bases and a maximum of 454. SGA coverage for this region was 87-fold.

Combining all 3 methods, we observed 1470 invariant positions (all calling the same reference base by all 3 methods) and 74 variant positions, 6 of which were called by all 3 methods (all calling the same major and minor allele/s), for a 95.6% concordance in overall calling. The remaining 68 variant positions were called only by a subset of methods.

Thirty-two sites were called by at least two methods (6 by all 3 and 26 by two of the three, with 24 called by 454 and TOPO-clone and 1 each by the other pairings). At all 32 sites, each method called the same major and minor allele/s. For the 30 sites called by both 454 and TOPO-clone sequencing, 20 showed no significant difference in frequency estimate (X2 < 3.84), 8 showed significant difference at the 5% level (X2 ≥ 3.84), and 2 were triallelic and not tested for concordance. Overall, the values were well correlated, with a Pearson’s correlation  = 0.79. Orthogonal regression of the frequency estimates showed no significant bias in frequency for either method, with a regression slope of 1.01 (95% c.i., 0.73 to 1.4), consistent with equal estimation of frequency by both methods (**Figure 2**).

For variants not detected by SGA, only 7 of 61 sites rejected the hypothesis of being randomly missed at the 5% level; with a standard Bonferroni 0.05/61 correct only two variants are significant. One of these is a clear 454 artifact (an ~11% variant with all variant calls on the same strand); further examination indicates that it results from sequencing of a PCR primer that was not trimmed correctly and that differs from the sample consensus at that position. The four sites found only by SGA all occur as 2 reads out of 87 (the sensitivity threshold) and are not seen at all in 454 or TOPO-clone sequencing. Sites found by 454 or TOPO-clone sequencing but not by SGA frequently reject the hypothesis of random read distribution. These results suggest that the high fold sequence coverage of 454 and TOPO-clone lead to increased sensitivity over SGA when equivalent coverage is not obtained.

Some amount of the non-concordance between the sequencing methods may be due to errors. Despite our strict cleaning, our sensitivity algorithms to first approximation assume independence of errors, which we know not to be the case (see **Figure S1** in **Text S1**). Some artifacts are method-specific and likely to generate method-specific variant calls, mostly at low frequency. The number of method-specific calls is proportional to the depth of coverage of the methods (28 for 454, 10 for clone, and 4 for SGA). This maps directly to sensitivity for rare variants. However, variants that are found by only one method are likely victims of the “winner’s curse”. There are likely to be many variants in the intrahost population that are below the frequency at which we are likely to observe them. If they are seen at all, they are likely to be seen only by a single method, and they are likely to have their frequencies substantially overestimated by that method (leading to a false expectation of seeing them by other methods). Further, since each sequence of TOPO-clone or SGA represents a single haplotype across the region (contrasted with 454 reads, which cover a single haplotype only over the length of a read), if we happened to randomly capture an excess of a rare haplotype with multiple variants, we would overestimate all of their frequencies.

Recently, Jordan *et al.* [28] published a comparison of clone-based sequencing and SGA sequencing and concluded that the methods provided similar results. Their comparison strategy was nearly orthogonal to ours. They sequenced a large number of different samples to low (12-15 fold) coverage and compared the average pairwise differences (APD) between sequences within each sample. They concluded that the amount of diversity measured by cloning and SGA are not significantly different. However, their low level of sequencing of each sample precludes a comparison of individual sites, as they have no expectation of finding the same low frequency variant by both methods. In our analysis, our deep sequence coverage by all three methods (454, clone, and SGA) allows us to compare sensitivity to detect and quantify individual mutations within a single sample, but since we have sequenced only a single sample to this depth, we cannot comment on the distribution of APD, as we have no measure of within-method variability.

| **Table S1. Assembly metrics for 89 samples assembled by AV454 where at least 70% of the**  **target region was covered at 10-fold.** | | | | | | | | | | | | | |
| --- | --- | --- | --- | --- | --- | --- | --- | --- | --- | --- | --- | --- | --- |
| Sample ID | Sample Type | No. Reads | No. Reads Aligned | Avg. Fold Sequence Coverage | Standard Deviation Fold Sequence Coverage | Percent of Genome Covered @ 10-fold | Percent of Genome Covered @ 50-fold | Percent of Genome Covered @ 100-fold | Percent of Genome Covered @ 200-fold | Percent of HXB2 Reference Target Region Covered by Largest Contig | HXB2 Start Nucleotide of Largest Contig | HXB2 Stop Nucleotide of Largest Contig | Percent of HXB2 Reference Target Region Covered by All Contigs |
| Average All Samples* |  | 9693 | 8016 | 241.8 | 152.5 | 94.6 | 74.0 | 58.7 | 39.0 | 96.3 | 908 | 9381 | 97.0 |
| V4141 | Infectious Clone | 40836 | 36010 | 959.1 | 948.1 | 99.7 | 97.3 | 95.7 | 91.7 | 100.0 | 780 | 9555 | 100.0 |
| V4136# | Acute | 29830 | 29003 | 724.4 | 375.9 | 100.0 | 98.4 | 96.5 | 92.9 | 100.0 | 767 | 9628 | 100.0 |
| V5282 | Acute | 3374 | 3295 | 112.6 | 47.6 | 100.0 | 86.5 | 63.6 | 4.5 | 100.0 | 759 | 9641 | 98.1 |
| V4394 | Chronic | 35112 | 20873 | 738.4 | 488.5 | 99.9 | 98.9 | 95.1 | 82.0 | 100.0 | 768 | 9556 | 100.0 |
| V4678# | Acute | 37803 | 14584 | 540.7 | 277.3 | 99.9 | 96.5 | 93.0 | 87.5 | 100.0 | 731 | 9607 | 100.0 |
| V4391 | Chronic | 13718 | 7535 | 213.3 | 102.6 | 99.9 | 97.6 | 86.3 | 52.7 | 100.0 | 761 | 9552 | 100.0 |
| V3048 | Chronic | 15656 | 12031 | 371.6 | 318.9 | 99.8 | 88.2 | 75.4 | 56.7 | 100.0 | 757 | 9592 | 91.8 |
| V4139# | Acute | 28963 | 27888 | 750.5 | 384.3 | 99.8 | 97.1 | 95.4 | 92.2 | 100.0 | 754 | 9566 | 100.0 |
| V4392 | Chronic | 12049 | 10410 | 245.6 | 120.5 | 99.8 | 95.5 | 88.1 | 64.7 | 100.0 | 766 | 9551 | 100.0 |
| V3021 | Chronic | 19661 | 17967 | 536.5 | 322.8 | 99.7 | 97.8 | 96.0 | 86.0 | 100.0 | 773 | 9551 | 100.0 |
| V4137# | Acute | 26103 | 25013 | 667.7 | 561.9 | 99.6 | 96.8 | 94.1 | 88.0 | 100.0 | 778 | 9557 | 100.0 |
| V4131 | Acute | 11586 | 11096 | 285.7 | 174.6 | 99.5 | 93.9 | 87.7 | 64.1 | 100.0 | 777 | 9551 | 100.0 |
| V4134 | Acute | 11516 | 10785 | 315.7 | 186.8 | 99.4 | 93.6 | 85.4 | 68.0 | 100.0 | 777 | 9551 | 100.0 |
| V3053 | Chronic | 10461 | 9727 | 327.8 | 164.6 | 99.3 | 95.3 | 91.0 | 73.8 | 100.0 | 759 | 9634 | 96.4 |
| V4119 | Acute | 13614 | 11658 | 325.5 | 226.3 | 99.1 | 91.1 | 84.9 | 61.9 | 100.0 | 773 | 9633 | 100.0 |
| V4123 | Acute | 9623 | 8358 | 219.4 | 122.6 | 99.1 | 91.3 | 83.2 | 55.3 | 100.0 | 765 | 9632 | 100.0 |
| V5261 | Acute | 3132 | 2777 | 104.0 | 52.7 | 98.9 | 75.1 | 64.8 | 1.7 | 100.0 | 775 | 9573 | 100.0 |
| V5276 | Acute | 2464 | 2427 | 89.4 | 44.3 | 98.7 | 75.0 | 46.4 | 0.0 | 100.0 | 777 | 9551 | 100.0 |
| V3120 | Chronic | 13478 | 12798 | 448.9 | 218.7 | 98.6 | 96.7 | 94.5 | 86.3 | 100.0 | 777 | 9552 | 100.0 |
| V3121 | Chronic | 5581 | 4558 | 134.8 | 95.1 | 98.6 | 77.8 | 56.7 | 23.2 | 100.0 | 756 | 9670 | 90.4 |
| V4140# | Acute | 12238 | 11642 | 299.7 | 209.3 | 98.5 | 93.6 | 87.7 | 64.3 | 100.0 | 777 | 9598 | 100.0 |
| V3047 | Chronic | 10902 | 8847 | 295.5 | 210.7 | 97.8 | 90.9 | 79.0 | 58.9 | 100.0 | 761 | 9551 | 100.0 |
| V3050 | Chronic | 3519 | 2581 | 88.0 | 68.7 | 97.4 | 59.4 | 32.2 | 11.6 | 100.0 | 777 | 9576 | 99.8 |
| V5245 | Acute | 1025 | 1012 | 36.1 | 18.4 | 97.4 | 21.3 | 0.0 | 0.0 | 100.0 | 777 | 9566 | 98.1 |
| V4122 | Acute | 8432 | 7586 | 202.2 | 103.2 | 97.3 | 91.2 | 82.1 | 52.7 | 100.0 | 779 | 9640 | 100.0 |
| V4135 | Acute | 14439 | 13769 | 405.7 | 251.2 | 97.2 | 93.7 | 89.4 | 71.9 | 100.0 | 777 | 9551 | 100.0 |
| V3027 | Chronic | 4145 | 3728 | 124.5 | 55.8 | 97.2 | 90.4 | 71.5 | 9.6 | 100.0 | 777 | 9558 | 100.0 |
| V3036 | Chronic | 3224 | 2288 | 72.8 | 37.2 | 97.0 | 74.6 | 20.6 | 0.0 | 100.0 | 776 | 9586 | 100.0 |
| V3114 | Chronic | 3657 | 3200 | 101.2 | 71.9 | 96.8 | 78.0 | 35.7 | 11.7 | 100.0 | 777 | 9636 | 100.0 |
| V3511 | Chronic | 3628 | 3518 | 120.2 | 110.1 | 96.3 | 54.8 | 44.4 | 26.2 | 100.0 | 738 | 9551 | 98.4 |
| V4507 | Viremic | 4329 | 3318 | 120.6 | 88.2 | 96.1 | 78.7 | 43.1 | 20.5 | 100.0 | 777 | 9574 | 99.9 |
| V3024 | Chronic | 10620 | 5528 | 176.9 | 107.4 | 96.0 | 81.5 | 70.7 | 43.3 | 100.0 | 765 | 9560 | 100.0 |
| V3527 | Chronic | 4476 | 4246 | 148.8 | 122.6 | 95.3 | 70.7 | 53.0 | 34.6 | 100.0 | 762 | 9575 | 90.0 |
| V5249 | Acute | 585 | 559 | 20.8 | 8.1 | 95.0 | 0.1 | 0.0 | 0.0 | 100.0 | 777 | 9562 | 100.0 |
| V3530 | Chronic | 4629 | 4095 | 93.3 | 67.8 | 94.5 | 69.3 | 39.9 | 9.4 | 100.0 | 757 | 9551 | 100.0 |
| V3529 | Chronic | 2691 | 2489 | 83.7 | 44.1 | 94.2 | 73.2 | 41.3 | 0.0 | 100.0 | 766 | 9561 | 100.0 |
| V3307 | Acute | 1947 | 1750 | 44.7 | 31.4 | 92.7 | 33.3 | 6.4 | 0.0 | 100.0 | 779 | 9551 | 100.0 |
| V4408 | Chronic | 2485 | 2360 | 87.3 | 64.2 | 91.8 | 69.0 | 31.6 | 8.6 | 100.0 | 743 | 9574 | 100.0 |
| V4388 | Chronic | 3642 | 2902 | 67.0 | 52.3 | 85.9 | 56.9 | 26.2 | 2.0 | 100.0 | 777 | 9552 | 100.0 |
| V5239 | Acute | 171 | 165 | 6.2 | 3.5 | 16.1 | 0.0 | 0.0 | 0.0 | 100.0 | 777 | 9575 | 100.0 |
| V4516 | Viremic | 1413 | 1266 | 44.7 | 24.9 | 93.3 | 36.9 | 2.5 | 0.0 | 100.0 | 782 | 9551 | 98.0 |
| V4121 | Acute | 9785 | 9090 | 234.0 | 134.9 | 98.5 | 93.3 | 85.2 | 50.7 | 100.0 | 777 | 9547 | 100.0 |
| V4393 | Chronic | 4273 | 2812 | 91.2 | 91.8 | 86.6 | 55.9 | 29.1 | 14.0 | 99.9 | 775 | 9546 | 100.0 |
| V4503 | Viremic | 1291 | 1031 | 35.2 | 18.3 | 91.8 | 20.6 | 0.0 | 0.0 | 99.7 | 777 | 9522 | 99.9 |
| V5279 | Acute | 4099 | 2416 | 90.6 | 53.5 | 81.2 | 74.7 | 54.2 | 0.2 | 99.6 | 810 | 9553 | 99.8 |
| V4390 | Chronic | 5833 | 4376 | 150.8 | 151.5 | 96.4 | 77.2 | 41.5 | 24.4 | 99.4 | 760 | 9500 | 99.7 |
| V3531 | Chronic | 5754 | 5462 | 183.1 | 86.6 | 99.7 | 93.1 | 79.6 | 42.9 | 99.4 | 764 | 9494 | 99.6 |
| V4474 | Chronic | 4251 | 3703 | 89.9 | 51.8 | 96.0 | 76.0 | 36.9 | 2.6 | 98.9 | 873 | 9592 | 97.6 |
| V3539 | Chronic | 2126 | 2053 | 75.5 | 38.1 | 95.0 | 76.7 | 16.9 | 0.0 | 98.7 | 777 | 9437 | 96.7 |
| V3306 | Acute | 8771 | 7368 | 199.1 | 126.2 | 92.3 | 83.7 | 75.0 | 50.3 | 98.5 | 778 | 9418 | 98.6 |
| V4120 | Acute | 9201 | 8280 | 213.6 | 110.7 | 99.1 | 92.8 | 83.3 | 56.0 | 98.4 | 763 | 9412 | 100.0 |
| V4470 | Chronic | 1457 | 1182 | 26.8 | 16.7 | 88.1 | 11.2 | 0.1 | 0.0 | 98.3 | 778 | 9399 | 98.2 |
| V4132 | Acute | 12080 | 11609 | 293.0 | 135.7 | 97.6 | 93.7 | 89.2 | 77.4 | 98.2 | 777 | 9397 | 100.0 |
| V3046 | Chronic | 7737 | 5254 | 178.4 | 84.4 | 99.8 | 93.8 | 82.5 | 39.9 | 98.2 | 768 | 9394 | 97.0 |
| V3533 | Chronic | 2716 | 2632 | 97.2 | 47.5 | 95.1 | 85.9 | 44.1 | 3.7 | 98.2 | 777 | 9391 | 98.4 |
| V4397 | Chronic | 9605 | 9369 | 345.5 | 153.4 | 98.6 | 96.0 | 92.3 | 83.5 | 97.9 | 727 | 9365 | 97.9 |
| V3032 | Chronic | 1359 | 1060 | 36.0 | 38.0 | 74.5 | 26.0 | 9.7 | 0.0 | 97.8 | 777 | 9359 | 98.0 |
| V3534 | Chronic | 6990 | 6500 | 193.8 | 95.0 | 97.9 | 91.6 | 80.9 | 49.8 | 97.8 | 777 | 9357 | 100.0 |
| V3115 | Chronic | 5152 | 4599 | 154.8 | 102.9 | 97.6 | 86.9 | 60.5 | 31.0 | 97.8 | 777 | 9354 | 97.5 |
| V3044 | Chronic | 18396 | 17411 | 593.3 | 392.8 | 99.3 | 95.2 | 90.4 | 75.3 | 97.7 | 743 | 9347 | 100.0 |
| V4478 | Chronic | 655 | 606 | 21.9 | 12.5 | 83.0 | 2.5 | 0.0 | 0.0 | 97.3 | 912 | 9446 | 97.3 |
| V3537 | Chronic | 1055 | 996 | 35.6 | 20.2 | 87.7 | 27.5 | 0.0 | 0.0 | 97.2 | 777 | 9309 | 97.3 |
| V4125 | Acute | 13851 | 12856 | 347.5 | 189.0 | 98.9 | 93.0 | 88.8 | 79.9 | 97.0 | 1044 | 9551 | 100.0 |
| V4489 | Viremic | 745 | 632 | 23.5 | 11.9 | 85.2 | 2.3 | 0.0 | 0.0 | 96.9 | 777 | 9282 | 95.6 |
| V4389 | Chronic | 5311 | 4869 | 172.7 | 175.0 | 88.5 | 58.9 | 49.3 | 39.3 | 96.4 | 1091 | 9551 | 98.3 |
| V3128 | Chronic | 34995 | 23759 | 842.9 | 530.2 | 100.0 | 99.6 | 97.3 | 90.3 | 96.4 | 782 | 9241 | 8.8 |
| V4676# | Acute | 16773 | 7162 | 227.6 | 141.8 | 96.9 | 87.3 | 73.8 | 57.8 | 96.0 | 858 | 9282 | 100.0 |
| V3039 | Chronic | 23454 | 22502 | 737.8 | 522.3 | 100.0 | 97.4 | 93.7 | 83.3 | 95.5 | 748 | 9153 | 100.0 |
| V3512 | Viremic | 12795 | 11570 | 333.4 | 156.3 | 99.0 | 95.8 | 91.7 | 77.1 | 95.4 | 883 | 9253 | 100.0 |
| V4506 | Viremic | 1029 | 902 | 31.9 | 20.6 | 79.5 | 16.8 | 0.0 | 0.0 | 93.9 | 778 | 9016 | 94.1 |
| V5258* | Acute | 1002 | 914 | 34.1 | 21.7 | 74.9 | 28.6 | 0.0 | 0.0 | 93.7 | 1331 | 9551 | 91.9 |
| V3538 | Chronic | 3280 | 2865 | 105.7 | 56.1 | 97.5 | 83.0 | 52.0 | 6.5 | 93.1 | 763 | 8950 | 87.5 |
| V3124* | Chronic | 5324 | 3943 | 148.9 | 117.1 | 80.8 | 73.5 | 53.4 | 24.7 | 84.8 | 744 | 8216 | 84.9 |
| V3515 | Viremic | 15133 | 13919 | 462.7 | 203.5 | 99.4 | 97.2 | 95.0 | 89.7 | 83.7 | 757 | 8121 | 100.0 |
| V4424* | Chronic | 5659 | 5387 | 246.6 | 174.7 | 74.8 | 65.6 | 54.5 | 46.8 | 78.9 | 2627 | 9551 | 79.0 |
| V4421* | Chronic | 2145 | 1974 | 89.5 | 46.9 | 73.1 | 63.2 | 38.9 | 0.0 | 78.8 | 2643 | 9551 | 78.9 |
| V3118* | Chronic | 10126 | 8127 | 382.0 | 269.0 | 76.7 | 72.2 | 68.4 | 55.3 | 76.6 | 2625 | 9347 | 79.1 |
| V3110 | Chronic | 15607 | 14274 | 472.0 | 324.6 | 98.8 | 90.4 | 83.7 | 75.2 | 61.3 | 780 | 6160 | 98.8 |
| V4124 | Acute | 14229 | 13287 | 350.2 | 176.2 | 98.6 | 93.9 | 89.4 | 81.2 | 59.2 | 4359 | 9589 | 59.1 |
| V3122* | Chronic | 3112 | 1620 | 61.8 | 44.3 | 72.7 | 54.6 | 18.1 | 0.0 | 58.6 | 4406 | 9550 | 91.6 |
| V3020 | Chronic | 8195 | 6035 | 152.2 | 114.5 | 99.0 | 82.8 | 61.0 | 26.8 | 58.2 | 771 | 5886 | 100.0 |
| V3111* | Chronic | 1350 | 1101 | 39.4 | 25.5 | 83.0 | 26.7 | 2.6 | 0.0 | 56.7 | 4395 | 9369 | 89.3 |
| V3117* | Chronic | 10614 | 9797 | 320.4 | 231.2 | 92.2 | 85.4 | 79.2 | 64.9 | 48.4 | 769 | 5026 | 92.0 |
| V4396* | Chronic | 6645 | 6173 | 232.2 | 125.0 | 89.0 | 83.3 | 76.8 | 55.9 | 45.6 | 757 | 4775 | 92.5 |
| V3521* | Chronic | 8629 | 7248 | 285.2 | 203.4 | 88.2 | 81.8 | 76.2 | 54.2 | 44.0 | 4395 | 8259 | 82.0 |
| V4404* | Chronic | 8728 | 8414 | 324.9 | 331.7 | 86.3 | 63.5 | 58.6 | 51.9 | 37.4 | 6272 | 9601 | 92.1 |
| V3009* | Chronic | 4936 | 3529 | 105.5 | 107.4 | 83.0 | 48.4 | 37.2 | 17.4 | 36.2 | 6211 | 9385 | 92.2 |
| V3007 | Chronic | 1925 | 1095 | 28.8 | 18.2 | 78.9 | 12.5 | 0.0 | 0.0 | 27.6 | 6630 | 9052 | 98.0 |
| V3010* | Chronic | 5117 | 4009 | 108.8 | 70.0 | 88.1 | 73.1 | 45.9 | 11.4 | 25.4 | 777 | 3009 | 92.9 |
| #Sample IDs for patient 9213 are highlighted in red; V4137 = day 0, V4136 = day 3, V4139 = day 59, V4140 = day 165, V4676 = day 476, V4678 = day 1543 *Partial genomes (i.e. less than 4 amplicons successfully amplified) are not included in averages | | | | | | | | | | | | | |

| **Table S2. Impact of alignment reference sequence on alignment quality.** | | | | | | | | | | | | |
| --- | --- | --- | --- | --- | --- | --- | --- | --- | --- | --- | --- | --- |
|  |  | **%Reads Aligned (p=0.091)1** | | |  | **% Bases Aligned (p=0.238)1** | | |  | **% Non-Multiple 3 Gaps in Reads (p<0.0001)1** | | |
| **Alignment Reference** |  | **Median** | **Min** | **Max** |  | **Median** | **Min** | **Max** |  | **Median** | **Min** | **Max** |
| *de novo* assembly |  | 92.2% | 39.3% | 99.6% |  | 93.8% | 54.0% | 99.5% |  | 0.0% | 0.0% | 0.1% |
| HXB2 |  | 89.7% | 38.5% | 98.5% |  | 92.4% | 53.7% | 98.8% |  | 0.2% | 0.0% | 0.4% |
| 1 significance determined by Wilcoxon Rank Sums | | | | | | | | | | | | |

| **Table S3A. Number nucleotide differences observed between Sanger consensus assembly versus *AssembleViral454 v1.0* consensus assembly** | | | | |
| --- | --- | --- | --- | --- |
| **Sample Time Point** | **Day 0** | **Day 3** | **Day 59** | **Day 165** |
| **Nucleotide Differences** | 0 | 0 | 1 | 5 + indel* |

| **Table S3B. Detail of observed nucleotide differences between Sanger consensus assembly and *AssembleViral454 v1.0* consensus assembly showing differences (highlighted in red) are due to variant diversity at these positions.** | | | | | | | | | | |
| --- | --- | --- | --- | --- | --- | --- | --- | --- | --- | --- |
| **Time** | **Day 0** | | **Day 3** | | **Day 59** | | | **Day 165** | | |
|  | **Sanger** | **454** | **Sanger** | **454** | **Sanger** | **454** | **Fraction 454 Reads Support Variant** | **Sanger** | **454** | **Fraction 454 Reads Support Variant** |
| **genome pos.** |  |  |  |  |  |  |  |  |  |  |
| 227 | G | G | G | G | G | G | 1.0G | A | G | .79G/.21A |
| 5870 | G | G | G | G | G | G | .97G/.03A | G | A | .63G/.37A |
| 5871 | C | C | C | C | C | C | .98C/.02T | T | C | .68C/.32T |
| 6836-6847 |  |  |  |  |  |  |  | Present | Deleted | Partially deleted* |
| 7310 | T | T | T | T | T | T | 1.0T | A | G | .56G/.38A/.05T/.01C |
| 8275 | A | A | A | A | G | A | .50G/.50A | G | G | .60G/.40A |
| 8478 | T | T | T | T | C | C | .51T/.49C | C | T | .61T/.39C |
| *There are no deletions called at 6836 because the consensus is the deletion type, but there are insertions. However, the counting of inserted bases is done such that it is difficult to accurately estimate the relative number of reads in each type. The 454 read data for day 165 show strong support for the existence in the intrahost population of both a long and a short haplotype across the 12 bp deletion (6836-6847) observed in the 454 assembly at day 165; however, it is difficult to precisely measure the frequencies of the two haplotypes due to the bias introduced by alignment of reads containing long indels relative to the reference. | | | | | | | | | | |

| **Table S4**. **CD8+ T cell epitopes restricted by subject 9213 that exhibit significantly greater sequence divergence (p<0.05) than non-epitope regions of their respective genes.** | | | | | | | | |
| --- | --- | --- | --- | --- | --- | --- | --- | --- |
| **Epitope** | **Sequence** | **Protein** | **Day 0** | **Day 3** | **Day 59** | **Day 165** | **Day 476** | **Day 1543** |
| A24-KW9 | KYKLKHIVW | p17 | <0.003 | 1.000 | 1.000 | 1.000 | <0.003 | <0.003 |
| A01-GY9 | GSEELRSLY | p17 | 1.000 | 1.000 | 1.000 | <0.003 | 1.000 | 0.108 |
| B44-LY9 | LYNTVATLY | p17 | 1.000 | 1.000 | 1.000 | 1.000 | 1.000 | <0.003 |
| B44-EV9 | EEKAFSPEV | p24 | 1.000 | 1.000 | 1.000 | 1.000 | 1.000 | 1.000 |
| A24-RL11 | RDYVDRFFKTL | p24 | 1.000 | 1.000 | 1.000 | 1.000 | 1.000 | 0.529 |
| B44-AW11 | AEQASQEVKNW | p24 | 1.000 | 1.000 | 1.000 | 1.000 | 1.000 | 0.721 |
| B44-EW9 | EEMNLPGRW | PR | 1.000 | 0.182 | 1.000 | 0.018 | 0.039 | 0.034 |
| B38-WI9 | WHLGQGVSI | Vif | 1.000 | 1.000 | <0.006 | <0.006 | <0.006 | 0.060 |
| Cw12-CC8 | CCFHCQVC | Tat | 1.000 | 1.000 | 1.000 | 1.000 | 1.000 | 0.579 |
| A01-IY9 | ISERILSTY | Rev | 1.000 | 0.247 | 1.000 | 0.493 | 0.137 | 0.616 |
| B44-AY10 | AENLWVTVYY | gp120 | 1.000 | 1.000 | <0.001 | 0.019 | 0.058 | 0.105 |
| A24-LY10 | LFCASDAKAY | gp120 | 1.000 | 1.000 | 0.512 | 0.230 | 1.000 | 0.622 |
| B38-MW9 | MHEDIISLW | gp120 | 1.000 | 1.000 | 1.000 | 0.113 | 1.000 | 0.754 |
| Cw04-SF9 | SFNCGGEFF | gp120 | 1.000 | 1.000 | 0.643 | 0.400 | 1.000 | 0.249 |
| A24-RL9 | RYLKDQQLL | gp41 | 1.000 | 1.000 | 1.000 | 0.432 | 0.225 | 0.582 |
| A01-RY9 | RRGWEVLKY | gp41 | 1.000 | 0.183 | 0.006 | 0.025 | 0.318 | 0.220 |
| A01-YT9 | YFPDWQNYT | Nef | 1.000 | 1.000 | 1.000 | 1.000 | 1.000 | 1.000 |
| A24-RW8 | RYPLTFGW | Nef | 1.000 | 1.000 | <0.006 | <0.006 | 0.200 | 0.394 |
| A01-WH10 | WRFDSRLAFH | Nef | <0.006 | <0.006 | 0.288 | 1.000 | 0.338 | 0.769 |
|  |  |  |  |  |  |  |  |  |
| 1. ***Green*** highlights epitopes in which divergence was significantly higher within the epitope than in flanking regions | | | | | | | | |
| of their respective gene. | |  |  |  |  |  |  |  |

**Table S5. Estimated rates of escape and reversion in CD8 T cell epitopes.**

| **CD8+ T cell**  **Epitope 1** | **Mutation type 2** | **Relative**  **Rate** | **Frequency of Escape Variant per Day 3**  **(std err)** | **Autologous**  **Sequence 4** | **Day**  **0**  **SFC 5** | **Day**  **59**  **SFC** | **Day 476 SFC** | **Day**  **661**  **SFC** |
| --- | --- | --- | --- | --- | --- | --- | --- | --- |
| **TARGETED CD8 EPITOPES:** | | | |  |  |  |  |  |
| **Vif B38-WI9** | **Escape** | **Fast** | **0.0987±0.0005** | **WHLGQGVSI** | **0** | **2744** | **3510** | **325** |
|  |  |  |  | **WHLGQGVAI** | **NT** | **2364** | **3440** | **279** |
|  |  |  |  | **WHLGQGVSV** | **NT** | **2114** | **3860** | **325** |
| **Nef A24-RW8** | **Escape** | **Fast** | **0.0976±0.0003** | **RYPLTFGW** | **0** | **2584** | **2790** | **130** |
|  |  |  |  | **RYPLTLGW** | **NT** | **174** | **0** | **72** |
|  |  |  |  | **RYPLMFGW** | **NT** | **924** | **640** | **107** |
|  |  |  |  | **RFPLTFGW** | **NT** | **194** | **220** | **0** |
| **Pol B44-EW9** | **Escape** | **Slow** | **0.0133±0.0007** | **EEINLPGRW** | **NT** | **814** | **2550** | **1820** |
|  |  |  |  | **EEIKLPGRW** | **NT** | **74** | **0** | **0** |
|  |  |  |  | **EDINLPGRW** | **NT** | **0** | **0** | **118** |
| **Env A01-RY9 (R794H)** | **Reversion** | **Slow** | **0.0087±0.0052** | **HRGWEILKY** | **NT** | **0** | **80** | **0** |
|  |  |  |  | **RRGWEILKY** | **NT** | **0** | **70** | **0** |
| **Env Cw04-SF9** | **Escape** | **Slow** | **0.0067±0.0005** | **SFNCGGEFF** | **0** | **0** | **190** | **72** |
| **Gag A01-GY9** | **Escape** | **Slow** | **0.0036±0.0004** | **GSEELRSLY** | **0** | **144** | **370** | **337** |
|  |  |  |  | **GSEELKSLY** | **NT** | **0** | **0** | **118** |
|  |  |  |  | **GSEELRSLF** | **NT** | **74** | **230** | **0** |
| **Env A24-RL9 (K593R)** | **Reversion** | **Slow** | **0.0026±0.0008** | **RYLRDQQLL** | **NT** | **0** | **0** | **0** |
|  |  |  |  | **RYLKDQQLL** | **0** | **0** | **0** | **84** |
| **Nef A01-YT9** | **None** |  |  | **YFPDWQNYT** | **0** | **0** | **70** | **0** |
| **Rev A01-IY9** | **None** |  |  | **ISAWVLSTR** | **NT** | **734** | **1330** | **0** |
| **Gag B44-AW11** | **None** |  |  | **AEQASQDVKNW** | **0** | **444** | **2120** | **394** |
|  |  |  |  |  |  |  |  |  |
| **NON-TARGETED CD8 EPITOPES:** | | | |  |  |  |  |  |
| **Nef A01-WH10 (F197S)** | **Reversion** | **Fast** | **0.0722±0.0046** | **WRFDSRLAFH** | **0** | **0** | **0** | **0** |
| **Env B44-AY10 (all)** | **Reversion** | **Fast** | **0.0887±0.0017** | **ADNWWVTVYY** | **NT** | **0** | **0** | **0** |
| **Gag A24-KW9 (I34V)** | **Reversion** | **Slow** | **0.0050±0.0013** | **QYRLKHVVW** | **0** | **0** | **0** | **0** |
| **Gag A24-KW9 (K28Q)** | **Reversion** | **Slow** | **0.0028±0.0007** | **QYRLKHVVW** | **0** | **0** | **0** | **0** |
| **Gag B44-LY9 (V82I)** | **Reversion** | **Slow** | **0.0027±0.0005** | **LYNTIAVLY** | **NT** | **0** | **0** | **0** |
| **Env B38-MW9** | **None** |  |  | **MQEDIISLW** | **0** | **0** | **0** | **0** |
| **Gag B44-EV9** | **None** |  |  | **EEKAFSPEV** | **0** | **0** | **0** | **0** |
| **Gag A24-RL11** | **None** |  |  | **RDYVDRFYKTL** | **0** | **0** | **0** | **0** |
| **Tat Cw12-CC8** | **None** |  |  | **CCLHCQKC** | **0** | **0** | **0** | **0** |
| **Env A24-LY10** | **None** |  |  | **LFCASDAKGY** | **0** | **0** | **0** | **0** |
|  |  |  |  |  |  |  |  |  |
| **NON-RESTRICTED, REVERTING MUTATIONS:** | | | | |  |  |  |  |
| **Nef B57-YY9 (I139T)** | **Reversion** | **Slow** | **0.0112±0.0016** | **YTPGPGTRY** | **N/A** | **N/A** | **N/A** | **N/A** |
| **Vif B57-IF9 (G37D)** | **Reversion** | **Slow** | **0.0119±0.0001** | **ISKKAKDWF** | **N/A** | **N/A** | **N/A** | **N/A** |
| **Pol B51-TI8 (I293T)** | **Reversion** | **Slow** | **0.0042±0.0001** | **TAFTIPST** | **N/A** | **N/A** | **N/A** | **N/A** |
| **Pol A03-ATK9 (T328I)** | **Reversion** | **Slow** | **0.0036±0.0006** | **AIFQSSMIK** | **N/A** | **N/A** | **N/A** | **N/A** |
| **Vpr A02-AL9 (I63T)** | **Reversion** | **Slow** | **0.0033±0.0005** | **AIIRTLQQL** | **N/A** | **N/A** | **N/A** | **N/A** |
| **Vif A03-RK10 (A20T)** | **Reversion** | **Slow** | **0.0033±0.0001** | **RIRAWKSLVK** | **N/A** | **N/A** | **N/A** | **N/A** |
| **Pol A03-QK9 (K435R)** | **Reversion** | **Slow** | **0.0015±0.0001** | **QIYAGIKVR** | **N/A** | **N/A** | **N/A** | **N/A** |

1 CD8+ T cell epitopes from LANL HIV “A list”; for escape, all variants within epitopes were assumed to contribute to escape and were included in the

estimate of escape rate; for reversion, the single specific reverting residue is shown in parentheses.

2 “Escape” mutations represent those that alter the defined optimal epitope suggesting a reduction in effective CD8+ T cell targeting; “reversion” mutations represent those that restore the defined consensus clade B sequence of the optimal epitope.

3 Rate of change estimated by best fit of the observed data to the HIV infection dynamics model of Asquith et al. by nonlinear least squares regression; the asymptotic standard error of the rate estimate is shown in parentheses.

4 Autologous and variant sequences of defined CD8 epitopes are listed. Amino acids in ‘*red’* indicate escape mutations away from the transmitted sequence that developed over time. Amino acids in ‘*blue*‘ indicate non-consensus mutations present in the Day 0 founder virus.

5 Magnitude of CD8+ T cell responses to defined CD8 epitopes in patient 9213 as measured by IFN-g ELISPOT assay, with responses reported as Spot Forming Cells (SFC) per million PBMC. 'NT' indicates that the respective peptide was not tested. 'N/A' indicates that the respective peptide is not applicable since it is not restricted by the HLA alleles expressed by subject 9213.

**Table S6. Haplotype Profiles of All Targeted CD8 Epitopes in Subject 9213.**

1. Consensus clade B (Cons B) sequence specific to the epitope of interest.

2. Percentage (%) of all high-quality reads for each haplotype presented.

3. Number (#) of high quality reads covering epitopic region of interest.

4. Day 0 sequence of each epitope. *Grey* bars denote the dominant haplotype per time point.

5. Day 0 residues in *blue* denote differences between consensus (Cons B) and the transmitted sequence.

6. Dots (.) denote amino acid residues matching the transmitted Day 0 sequence.

7. Haplotypes in *red* denote sequences containing mutations not present at Day 0 (D0).

8. Haplotypes in *blue* denote mutations consistent with reversion to clade B consensus (Cons B).

9. The L890F mutation in *red* is likely selected by the overlapping targeted A01-GY9 epitope.

**Table S7. Haplotype Profiles of All Non-Targeted CD8 Epitopes in Subject 9213.**

**Table S8. Haplotype Profiles of Non-Restricted and Reverting CD8 Epitopes.**

| **Table S9**. Non-default parameter settings for *Mosaik* *v1.0.1388* alignments | | | |
| --- | --- | --- | --- |
|  |  |  |  |
| **For *Mosaik*Build Module:** | |  |  |
|  |  |  |  |
| Parameter | Description | Setting | Comments |
| -st | sequencing technology | 454 |  |
| -tn | number of N allowed in a read | 500 | high setting prevents reads with large number of “N’s” or significant diversity from the reference from being eliminated |
|  |  |  |  |
| **For *Mosaik*Aligner Module:** | |  |  |
|  |  |  |  |
| Parameter | Description | Setting | Comments |
| -hs | hash size |  |  |
| -act | alignment candidate threshold | 10 |  |
| -mmp | maximum mismatch percentage | 15 |  |
| -minp | mimimum percentage of read length aligned | 0.25 |  |
| -mmal | errors counted using aligned read length | invoked |  |
| -m | determines reads kept in alignment | unique |  |
| -gop | gap opening penalty | 30 | only invoked for alignments following homopolymer correction and at completion of all read clean-up steps |
|  |  |  |  |
|  |  |  |  |


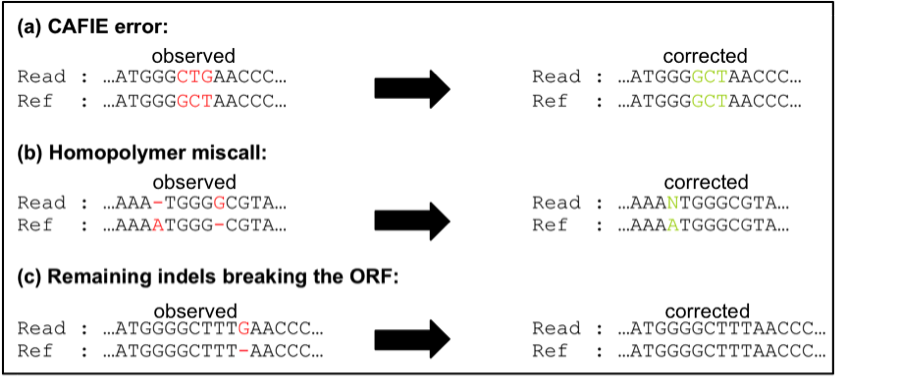


**Figure S1**. **Overview of read clean-up process implemented in *RC454 v1.0* algorithm.** (a) Carry Forward Incomplete Extension (CAFIE) errors inherent to 454 pyrosequencing are identified (i.e. region of read where the last base of a homopolyer run is found in the next flow run) and corrected by placement of last base of homopolymer run in correct position. (b) Homopolymer undercalls and overcalls are corrected by adding an N if there is a deletion in the read relative to the reference and by removal of the base from the read if the reference does not support the read overcall. (c) Remaining InDels are evaluated to determine if they are supported by protein coding information and read insertions are removed if they break frame in any gene and do not occur in multiples of three within 10 bases upstream or downstream of the insertion being evaluated.

**
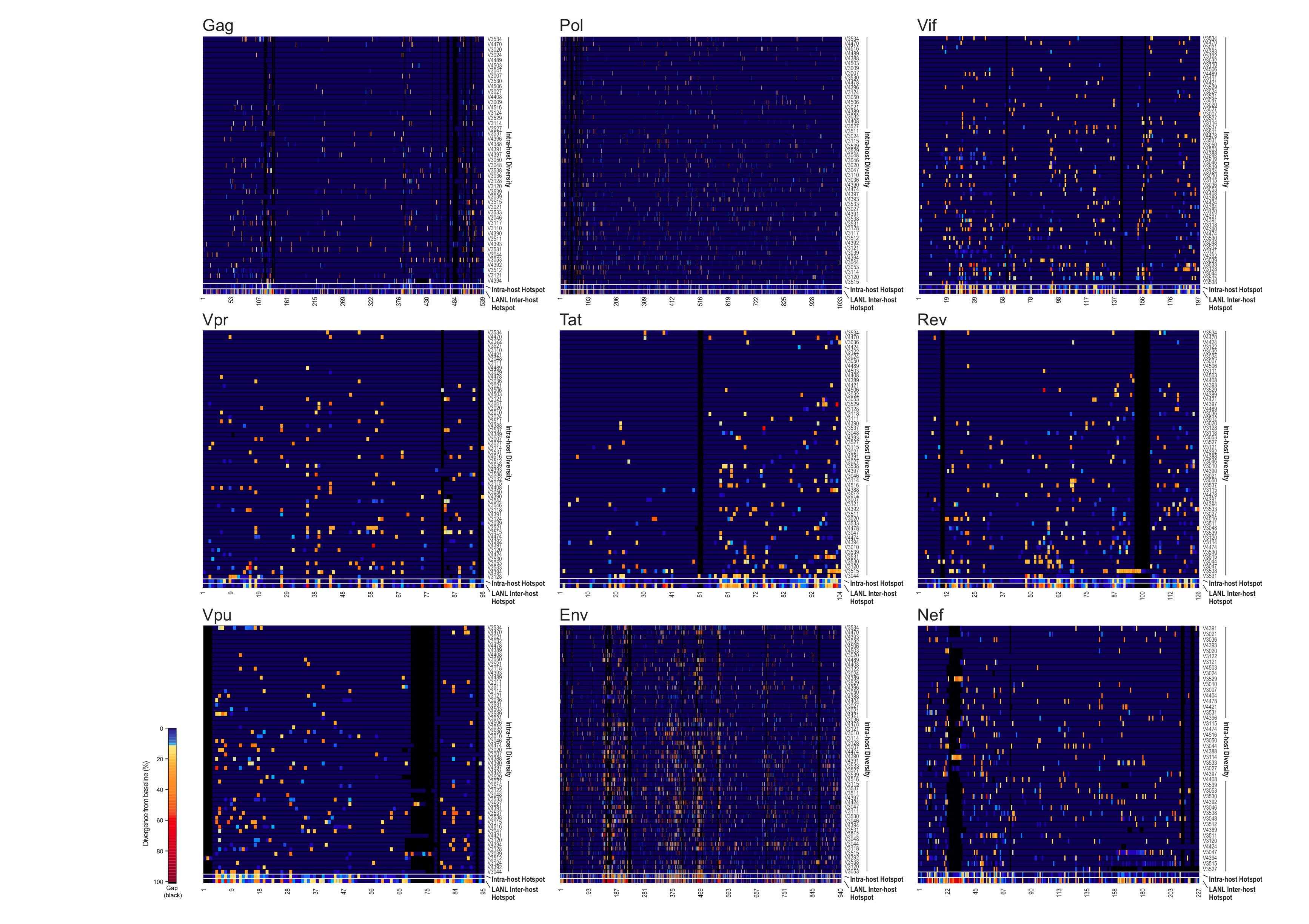
**

**Figure S2. Intra-host and inter-host viral diversity in HIV-1 infected patients.** Heat maps for each HIV-1 protein illustrate sites exhibiting amino acid sequence diversity in HIV-1B infected subjects (V#’s in each row correspond to individual subjects). Amino acid residue positions for each protein are shown at the bottom of each heat map. Intra-host diversity for each subject sequenced by 454 (large upper block of each panel) is plotted as the percentage of amino acids differing from the consensus call for each respective residue. At the bottom of each panel an ‘Intra-host Hotspot’ bar reflects the percentage of intra-host samples that exhibit detectable variation at each respective position. The ‘LANL Inter-host Hotspot’ bar reflects global inter-host diversity calculated as the percentage of genomes that have a non-majority consensus call at the respective residue in the genome. Inter-host diversity is calculated from a curated alignment of HIV-1B sequences for each gene obtained from the LANL HIV Sequence Database (<http://www.hiv.lanl.gov/>).

**SUPPLEMENTARY REFERENCES:**

1. Streeck H, Lichterfeld M, Alter G, Meier A, Teigen N, et al. (2007) Recognition of a defined region within p24 gag by CD8+ T cells during primary human immunodeficiency virus type 1 infection in individuals expressing protective HLA class I alleles. J Virol 81: 7725-7731.

2. Christopherson C, Sninsky J, Kwok S (1997) The effects of internal primer-template mismatches on RT-PCR: HIV-1 model studies. Nucleic Acids Res 25: 654-658.

3. Trujillo JR, Wang WK, Lee TH, Essex M (1996) Identification of the envelope V3 loop as a determinant of a CD4-negative neuronal cell tropism for HIV-1. Virology 217: 613-617.

4. Yu Q, Ryan EM, Allen TM, Birren BW, Henn MR, et al. (2011) PriSM: a primer selection and matching tool for amplification and sequencing of viral genomes. Bioinformatics 27: 266-267.

5. Harismendy O, Frazer K (2009) Method for improving sequence coverage uniformity of targeted genomic intervals amplified by LR-PCR using Illumina GA sequencing-by-synthesis technology. Biotechniques 46: 229-231.

6. Lennon NJ, Lintner RE, Anderson S, Alvarez P, Barry A, et al. (2010) A scalable, fully automated process for construction of sequence-ready barcoded libraries for 454. Genome Biol 11: R15.

7. St Louis DC, Gotte D, Sanders-Buell E, Ritchey DW, Salminen MO, et al. (1998) Infectious molecular clones with the nonhomologous dimer initiation sequences found in different subtypes of human immunodeficiency virus type 1 can recombine and initiate a spreading infection in vitro. J Virol 72: 3991-3998.

8. Liu Y, Woodward A, Zhu H, Andrus T, McNevin J, et al. (2009) Preinfection human immunodeficiency virus (HIV)-specific cytotoxic T lymphocytes failed to prevent HIV type 1 infection from strains genetically unrelated to viruses in long-term exposed partners. J Virol 83: 10821-10829.

9. Bernardin F, Herring BL, Peddada L, Delwart EL (2003) Primary infection of a male plasma donor with divergent HIV variants from the same source followed by rapid fluctuations in their relative frequency and viral recombination. AIDS Res Hum Retroviruses 19: 1009-1015.

10. Altfeld M, Allen TM, Yu XG, Johnston MN, Agrawal D, et al. (2002) HIV-1 superinfection despite broad CD8+ T-cell responses containing replication of the primary virus. Nature 420: 434-439.

11. Palmer S, Kearney M, Maldarelli F, Halvas EK, Bixby CJ, et al. (2005) Multiple, linked human immunodeficiency virus type 1 drug resistance mutations in treatment-experienced patients are missed by standard genotype analysis. J Clin Microbiol 43: 406-413.

12. Pevzner PA, Tang H, Waterman MS (2001) An Eulerian path approach to DNA fragment assembly. Proc Natl Acad Sci U S A 98: 9748-9753.

13. Zerbino DR, Birney E (2008) Velvet: algorithms for de novo short read assembly using de Bruijn graphs. Genome Res 18: 821-829.

14. Li R, Li Y, Kristiansen K, Wang J (2008) SOAP: short oligonucleotide alignment program. Bioinformatics 24: 713-714.

15. Pop M, Phillippy A, Delcher AL, Salzberg SL (2004) Comparative genome assembly. Brief Bioinform 5: 237-248.

16. Jaffe DB, Butler J, Gnerre S, Mauceli E, Lindblad-Toh K, et al. (2003) Whole-genome sequence assembly for mammalian genomes: Arachne 2. Genome Res 13: 91-96.

17. Batzoglou S, Jaffe DB, Stanley K, Butler J, Gnerre S, et al. (2002) ARACHNE: a whole-genome shotgun assembler. Genome Res 12: 177-189.

18. Garber M, Zody MC, Arachchi HM, Berlin A, Gnerre S, et al. (2009) Closing gaps in the human genome using sequencing by synthesis. Genome Biol 10: R60.

19. Birney E, Clamp M, Durbin R (2004) GeneWise and Genomewise. Genome Res 14: 988-995.

20. Brockman W, Alvarez P, Young S, Garber M, Giannoukos G, et al. (2008) Quality scores and SNP detection in sequencing-by-synthesis systems. Genome Res 18: 763-770.

21. Wang C, Mitsuya Y, Gharizadeh B, Ronaghi M, Shafer RW (2007) Characterization of mutation spectra with ultra-deep pyrosequencing: application to HIV-1 drug resistance. Genome Res 17: 1195-1201.

22. Altshuler D, Pollara VJ, Cowles CR, Van Etten WJ, Baldwin J, et al. (2000) An SNP map of the human genome generated by reduced representation shotgun sequencing. Nature 407: 513-516.

23. Zagordi O, Klein R, Daumer M, Beerenwinkel N (2010) Error correction of next-generation sequencing data and reliable estimation of HIV quasispecies. Nucleic Acids Res 38: 7400-7409.

24. Asquith B, Edwards CT, Lipsitch M, McLean AR (2006) Inefficient cytotoxic T lymphocyte-mediated killing of HIV-1-infected cells in vivo. PLoS Biol 4: e90.

25. Goonetilleke N, Liu MK, Salazar-Gonzalez JF, Ferrari G, Giorgi E, et al. (2009) The first T cell response to transmitted/founder virus contributes to the control of acute viremia in HIV-1 infection. J Exp Med 206: 1253-1272.

26. Altschul SF, Madden TL, Schaffer AA, Zhang J, Zhang Z, et al. (1997) Gapped BLAST and PSI-BLAST: a new generation of protein database search programs. Nucleic Acids Res 25: 3389-3402.

27. Tan C, Iglewicz B (1999) Measurement methods comparisons and linear statistical relationships. Technometrics 41: 192-201.

28. Jordan MR, Kearney M, Palmer S, Shao W, Maldarelli F, et al. (2010) Comparison of standard PCR/cloning to single genome sequencing for analysis of HIV-1 populations. J Virol Methods 168: 114-120.
